# High-resolution visualization and assessment of basal and OXPHOS-induced mitophagy in H9c2 cardiomyoblasts

**DOI:** 10.1080/15548627.2023.2230837

**Published:** 2023-07-05

**Authors:** Gustav Godtliebsen, Kenneth Bowitz Larsen, Zambarlal Bhujabal, Ida S. Opstad, Mireia Nager, Abhinanda R. Punnakkal, Trine B. Kalstad, Randi Olsen, Trine Lund, Dilip K. Prasad, Krishna Agarwal, Truls Myrmel, Asa Birna Birgisdottir

**Affiliations:** a Department of Clinical Medicine, UiT-The Arctic University of Norway, Tromsø, Norway; b Department of Medical Biology, UiT-The Arctic University of Norway, Tromsø, Norway; c Department of Physics and Technology, UiT-The Arctic University of Norway, Tromsø, Norway; d Division of Cardiothoracic and Respiratory Medicine, UiT-The Arctic University of Norway, Tromsø, Norway; e Department of Computer Science, UiT-The Arctic University of Norway, Tromsø, Norway

**Keywords:** CLEM, deep learning, lysosomes, mitochondria, quality control, SIM

## Abstract

Mitochondria are susceptible to damage resulting from their activity as energy providers. Damaged mitochondria can cause harm to the cell and thus mitochondria are subjected to elaborate quality-control mechanisms including elimination via lysosomal degradation in a process termed mitophagy. Basal mitophagy is a house-keeping mechanism fine-tuning the number of mitochondria according to the metabolic state of the cell. However, the molecular mechanisms underlying basal mitophagy remain largely elusive. In this study, we visualized and assessed the level of mitophagy in H9c2 cardiomyoblasts at basal conditions and after OXPHOS induction by galactose adaptation. We used cells with a stable expression of a pH-sensitive fluorescent mitochondrial reporter and applied state-of-the-art imaging techniques and image analysis. Our data showed a significant increase in acidic mitochondria after galactose adaptation. Using a machine-learning approach we also demonstrated increased mitochondrial fragmentation by OXPHOS induction. Furthermore, super-resolution microscopy of live cells enabled capturing of mitochondrial fragments within lysosomes as well as dynamic transfer of mitochondrial contents to lysosomes. Applying correlative light and electron microscopy we revealed the ultrastructure of the acidic mitochondria confirming their proximity to the mitochondrial network, ER and lysosomes. Finally, exploiting siRNA knockdown strategy combined with flux perturbation with lysosomal inhibitors, we demonstrated the importance of both canonical as well as non-canonical autophagy mediators in lysosomal degradation of mitochondria after OXPHOS induction. Taken together, our high-resolution imaging approaches applied on H9c2 cells provide novel insights on mitophagy during physiologically relevant conditions. The implication of redundant underlying mechanisms highlights the fundamental importance of mitophagy.

**Abbreviations:** ATG: autophagy related; ATG7: autophagy related 7; ATP: adenosine triphosphate; BafA1: bafilomycin A_1_; CLEM: correlative light and electron microscopy; EGFP: enhanced green fluorescent protein; MAP1LC3B: microtubule associated protein 1 light chain 3 beta; OXPHOS: oxidative phosphorylation; PepA: pepstatin A; PLA: proximity ligation assay; PRKN: parkin RBR E3 ubiquitin protein ligase; RAB5A: RAB5A, member RAS oncogene family; RAB7A: RAB7A, member RAS oncogene family; RAB9A: RAB9A, member RAS oncogene family; ROS: reactive oxygen species; SIM: structured illumination microscopy; siRNA: short interfering RNA; SYNJ2BP: synaptojanin 2 binding protein; TEM: transmission electron microscopy; TOMM20: translocase of outer mitochondrial membrane 20; ULK1: unc-51 like kinase 1.

## Introduction

Mitochondria are the main energy producing organelles in eukaryotic cells and are also central in the control of redox homeostasis, Ca^2+^ signaling, iron metabolism, innate immunity and programmed cell death [1–5]. In most cell types, mitochondria are arranged in highly dynamic networks, controlled by constant fusion and fission events [6] driven by mitochondria movements along the cytoskeleton [7]. Events such as cell cycle progression, cellular differentiation, oxidative stress, metabolic perturbation and engagement in programmed cell death, all lead to significant alterations in the architecture of the mitochondrial network [8].

Mitochondria are dependent on oxygen for energy production in the form of adenosine triphosphate (ATP) through oxidative phosphorylation (OXPHOS). Reactive oxygen species (ROS) are formed as by-products during OXPHOS and thus mitochondria are susceptible to mitochondrial DNA mutations and protein misfolding that can ultimately lead to mitochondrial damage [9]. Damaged mitochondria result in energy-generation defects, the increased production of harmful reactive oxygen species and can trigger programmed cell death when the damage is beyond repair. Hence, mitochondria are subjected to elaborate quality control mechanisms [10]. Damaged mitochondria can be selectively eliminated by one such mechanism, termed mitophagy, dependent on the autophagy machinery [11–13]. Mitophagy is often preceded by fission of damaged mitochondria (or parts of mitochondria) from the mitochondrial network, followed by sequestration by a double‐membrane‐bound autophagosome and culminates in fusion with a lysosome where the mitochondria are degraded by resident acidic hydrolases. There are multiple signaling pathways that govern autophagosome engulfment of damaged mitochondria [14].

Macroautophagy/autophagy initiation in mammalian cells is driven by the ULK1 (unc-51 like kinase 1) protein kinase complex that phosphorylates and activates key downstream mediators involved in autophagosome formation [15]. Activation and recruitment of the ULK1 complex has been implicated in mitophagy [16–19]. Autophagosome formation involves lipidation of the Atg8 (autophagy-related 8)-family proteins such as MAP1LC3B (microtubule associated protein 1 light chain 3 beta). Here, Atg8-family proteins are conjugated to phosphatidylethanolamine (PE) in the phagophore membrane, representing one of the key molecular signatures of canonical autophagy [20]. Atg8-family protein lipidation is a multistep process driven by the enzymatic activity of core autophagy proteins: the E1-like ATG7 (autophagy related 7), the E2-like ATG3 (autophagy related 3) and the E3-like ATG12–ATG5-ATG16L1 complex [21–23].

Selected cargo such as mitochondria destined for degradation are connected to the forming autophagosome through binding to Atg8-family proteins in a ubiquitin dependent or independent manner [10]. Intriguingly, non-canonical pathways, independent of ATG7 and Atg8-family protein lipidation, have also been described for lysosomal clearance of damaged mitochondria. These include alternative autophagy [24,25], microautophagy involving formation of small mitochondria derived vesicles (MDVs) [26] and degradation through the endo-lysosomal pathway involving the endosomal small GTPase RAB5A (RAB5A, member RAS oncogene family) [27]. Furthermore, non-canonical mitophagy described in mouse cardiomyocytes depends on ULK1 and the small GTPase RAB9A (RAB9A, member RAS oncogene family) [28]. Interestingly, mitochondria and lysosomes can also directly interact through the small GTPase RAB7A (RAB7A, member RAS oncogene family) [29].

The most studied ubiquitin-dependent mitophagy is known as PINK-PRKN-dependent mitophagy, orchestrated by the enzyme 3 (E3) ubiquitin ligase PRKN (parkin RBR E3 ubiquitin protein ligase) and the protein kinase PINK1 (PTEN induced putative kinase 1) [30]. Pioneering work for elucidation of PRKN-mediated mitophagy relied on induction of mitophagy by using cytotoxic agents targeting mitochondria, resulting in membrane potential dissipation of the entire network and loss of most of the mitochondria [31,32]. In contrast, basal mitophagy is considered a house-keeping mechanism where mitochondrial content is fine-tuned depending on the metabolic state of the cell [9,14]. Thus, use of mitochondria depolarizing agents is not optimal to simulate physiological situations. Notably, the molecular mechanisms governing basal level of mitophagy in cells under physiological conditions remain mostly elusive.

In this work we set out to visualize and assess mitophagy in H9c2 cardiomyoblasts during normal culture conditions and after OXPHOS induction by galactose adaptation. We exploited H9c2 cells expressing pH-sensitive tandem mCherry-enhanced green fluorescent protein (EGFP) fluorescent mitochondrial reporters and applied state-of-the-art imaging methods for a detailed characterization of mitochondrial fragments within acidic compartments. Our results provide novel insights into the dynamics and regulation of lysosomal degradation of mitochondria in physiologically relevant settings.

## Results

### H9c2 cells with a pH-sensitive fluorescent mitochondrial reporter display induced formation of acidic mitochondria when adapted to galactose

**Figure 1.**
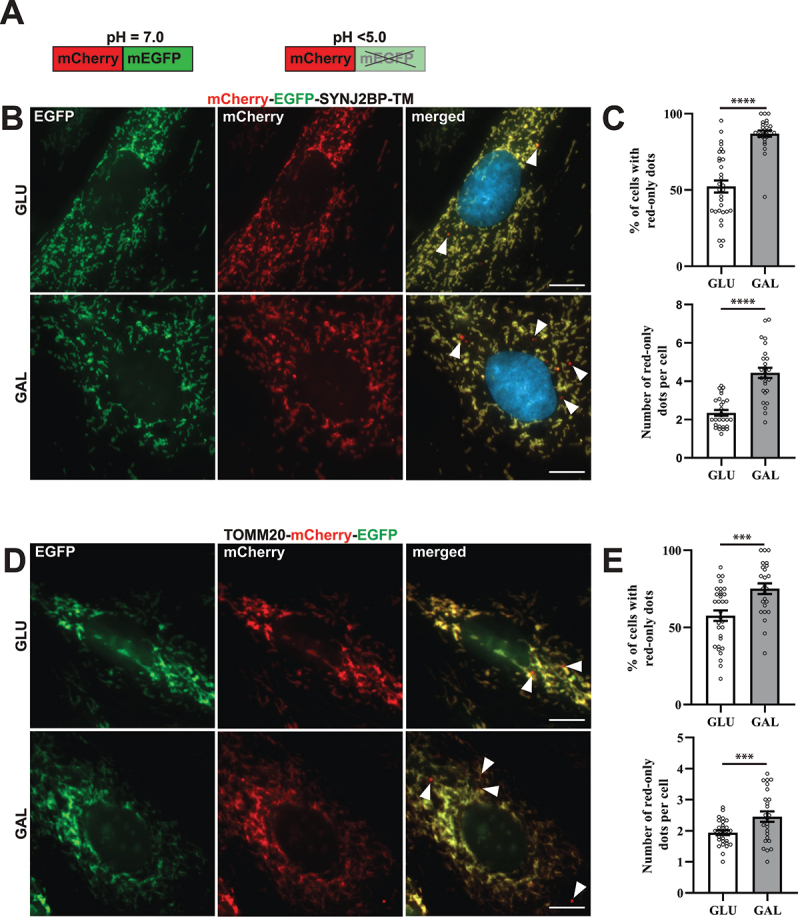
Reporter H9c2 cells display an increase in acidic mitochondria during galactose adaption. (A) An illustration of the pH-sensitive tandem fluorescent reporter mCherry-EGFP at different pH levels (red and green at neutral pH and red-only at acidic pH). (B) Representative widefield fluorescence microscopy images of H9c2 cells with a stable expression of the mCherry-EGFP-SYNJ2BP-TM reporter grown in glucose (GLU) or adapted to galactose (GAL) media. (C) Quantification of the percentage of cells containing red-only dots and quantification of the number of red-only dots per cell in cells with red-only dots in glucose vs galactose media for cells with the mCherry-EGFP-SYNJ2BP-TM reporter. (D) Representative widefield fluorescence microscopy images of H9c2 cells with a stable expression of the TOMM20-mCherry-EGFP reporter grown in glucose (GLU) or adapted to galactose (GAL) media. (E) Quantification of the percentage of cells containing red-only dots and quantification of the number of red-only dots per cell in cells with red only dots in glucose vs galactose media for cells with the TOMM20-mCherry-EGFP reporter. Data presented in (C) and (E) is shown as mean ± SEM from 3 independent experiments, with more than 100 cells in each condition. The individual datapoints are per frame cell averages.

Note: * *p* < 0.05, ** *p* < 0.01, *** *p* < 0.001 and **** *p* < 0.0001. Scale bar: 10 μm.

In order to monitor lysosomal degradation of mitochondria in rat cardiac myoblasts (H9c2) we established stable cell lines with constitutive expression of a fluorescent (mCherry-EGFP) tandem-tagged trans-membrane (TM) domain of the outer mitochondrial membrane protein SYNJ2BP/OMP25 (synaptojanin 2 binding protein) [33–35] or tandem tagged full-length TOMM20 (translocase of the outer mitochondrial membrane 20) protein. The mitochondria thus display both green (EGFP) and red (mCherry) fluorescence and appear yellow in merged images of the green and the red channel during fluorescence imaging of the cells. The EGFP fluorescence is quenched at low pH while the mCherry fluorescence is acid stable (Figure 1A) [36]. Therefore, during imaging, mitochondria or parts of mitochondria in acidic compartments (late endosomes or lysosomes) appear as red-only structures in merged images. Under normal culture conditions, red-only dots were easily detected by fluorescence microscopy of stably transfected H9c2 cells, thus enabling monitoring of basal levels of lysosomal acidification of engulfed mitochondria (Figure 1B). In order to investigate the effect of a metabolic shift on mitochondria degradation, the cells were adapted to galactose in glucose-free growth medium for a minimum of 7 days. In this way the cells become mostly dependent on OXPHOS for ATP production [37]. As a control, the cells were kept in normal high glucose containing media for the same time-period and propagated simultaneously. The cells were then fixed and imaged by fluorescence microscopy (Figure 1B). Image analysis software was used to assess the number of cells containing red-only mitochondria as well as the number of red-only dots per cell (Figure S1A). Our data demonstrate that during normal culture conditions approximately 50% of the mCherry-EGFP-SYNJ2BP-TM cells displayed acidic mitochondria (Figure 1C). Interestingly, a significant increase in the number of cells with acidic mitochondria was detected in the galactose-adapted cells (approximately 90%) with an almost a two-fold increase in the number of red-only dots per cell (Figure 1C). Similarly, the TOMM20-mCherry-EGFP H9c2 cells displayed an increase in the number of red-only dots per cell when adapted to galactose (Figure 1D,E). In comparison, 16 h of hypoxia (0.3% O_2_), a well-known inducer of mitophagy [38], resulted in an equivalent increase in the number of red-only structures per cell (Figure S1B). Furthermore, using transient transfection of the pH-sensitive mt-Keima probe targeted to the mitochondrial matrix [39,40], we detected a significant increase in acidic mitochondria per cell in galactose adapted H9c2 cells (Figure S1C). To summarize, galactose adaptation of H9c2 cells induced lysosomal engulfment of mitochondria visualized by different pH-sensitive mitochondria targeted probes.

### Galactose adaptation of H9c2 cells results in more fragmented mitochondria morphology, elevated mitochondrial respiration and higher susceptibility to mitochondrial damage.

**Figure 2.**
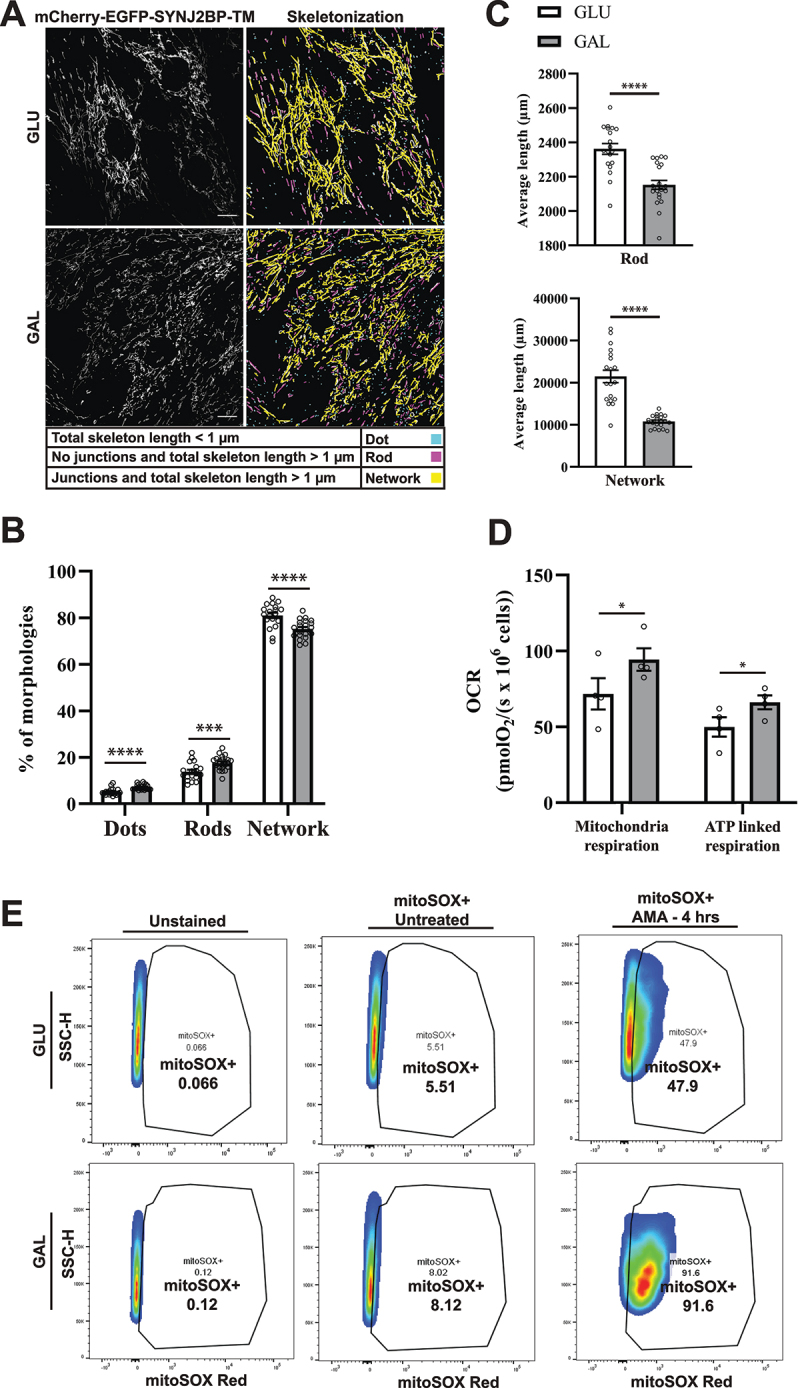
Mitochondria morphology analysis, mitochondrial respiration and ROS measurements indicate more fragmented mitochondria and OXPHOS dependance after galactose adaptation. (A) Morphological analysis of confocal images of cells grown in glucose vs galactose media. A representative skeletonization mask is depicted for each culture condition with the different morphology classes indicated in different colors. Rules for morphology classes are displayed at the bottom. (B) Overview of the distribution of mitochondria morphology classes in percentage for the two different growth conditions. The distribution for glucose vs galactose adaption is 5.16% vs 7.14% for dots, 13.79% vs 17.58% for rods and 81.04% vs 75.28% for network. (C) Quantification of the average length of mitochondria in the rod and network morphology classes for cells grown in glucose vs galactose. (D) High-resolution respirometry performed in an Oxygraph-2k (Oroboros Instruments). Oxygen consumption rate, OCR (pmolO_2_/s × 10^6^ cells) of cells grown under normal conditions (high glucose) or adapted to galactose was corrected for residual oxygen consumption (ROX) and normalized to the cell number per ml in the chambers. The values displayed in the graph are from four independent experiments ± SEM, indicating mitochondrial respiration (basal respiration minus ROX) and ATP-linked respiration (basal respiration minus proton leak), respectively. (E) Flow cytometry analysis of H9c2 cells grown in normal media with glucose or adapted to galactose. The antimycin A (AMA) treated/untreated cells were stained with mitoSOX Red and compared with unstained cells as a negative control. During normal culture conditions, antimycin A treatment resulted in approximately 47% mitoSOX Red positive cells, while in galactose 91% of the cells were mitoSOX Red positive. The data depicted represent one of three independent experiments. Data presented in (B) and (C) is shown as mean ± SEM of 20 fields of view per condition. The individual datapoints are per frame cell averages.

Note: * *p* < 0.05, ** *p* < 0.01, *** *p* < 0.001 and **** *p* < 0.0001. Scale bar: 10 μm.

Degradation of mitochondria is commonly preceded by mitochondrial fission or fragmentation [41,42]. We performed computational image analysis of mitochondrial morphology in the tandem-tagged H9c2 cells using machine learning. This enabled classification and quantification of the mitochondria morphology as networks, rods or dots as described previously [43,44]. We used confocal images of fixed mCherry-EGFP-SYNJ2BP-TM H9c2 cells grown under normal culture conditions or adapted to galactose (Figure 2A). Our computational analysis revealed that for both growth conditions, most of the mitochondria were in a network but for the galactose adapted cells the percentage was significantly lower and there were more mitochondria classified as rods and dots (Figure 2B). Furthermore, the average length of rod-shaped mitochondria as well as mitochondrial networks was significantly shorter in the galactose adapted cells (Figure 2C). Thus, our morphology analysis showed more fragmented mitochondria in galactose adapted cells. To demonstrate that galactose adaptation of the cells in fact induces OXPHOS, we characterized mitochondrial function by performing high-resolution respirometry using an Oxygraph-2k (Oroborus Instruments). Our results showed that cells adapted to galactose displayed higher mitochondrial respiration and higher ATP-linked respiration in comparison to cells under normal culture conditions, indicative of induced OXPHOS (Figure 2D). For investigation of the level of mitochondrial ROS production we applied the mitochondria-targeted superoxide indicator MitoSOX Red in H9c2 cells in glucose or adapted to galactose. This indicator gives rise to a fluorescent signal in the presence of mitochondria superoxide [45]. To measure mitochondrial ROS induction, we subjected these cells to antimycin A treatment for 4 h. Live confocal imaging of the cells (Figure S2) and Flow cytometry (Figure 2E) revealed substantially higher mitochondrial ROS production in galactose adapted cells in comparison to cells in glucose after antimycin A treatment. This demonstrates that the cells in galactose have become more dependent on their mitochondria and thus are more susceptible to mitochondrial toxicants [46]. Taken together, these results are consistent with OXPHOS-induced degradation of mitochondria after galactose adaptation as indicated by the increase in red-only structures.

### Red-only structures stain positive for markers of the different mitochondrial compartments but are devoid of mitochondrial membrane potential.

**Figure 3.**
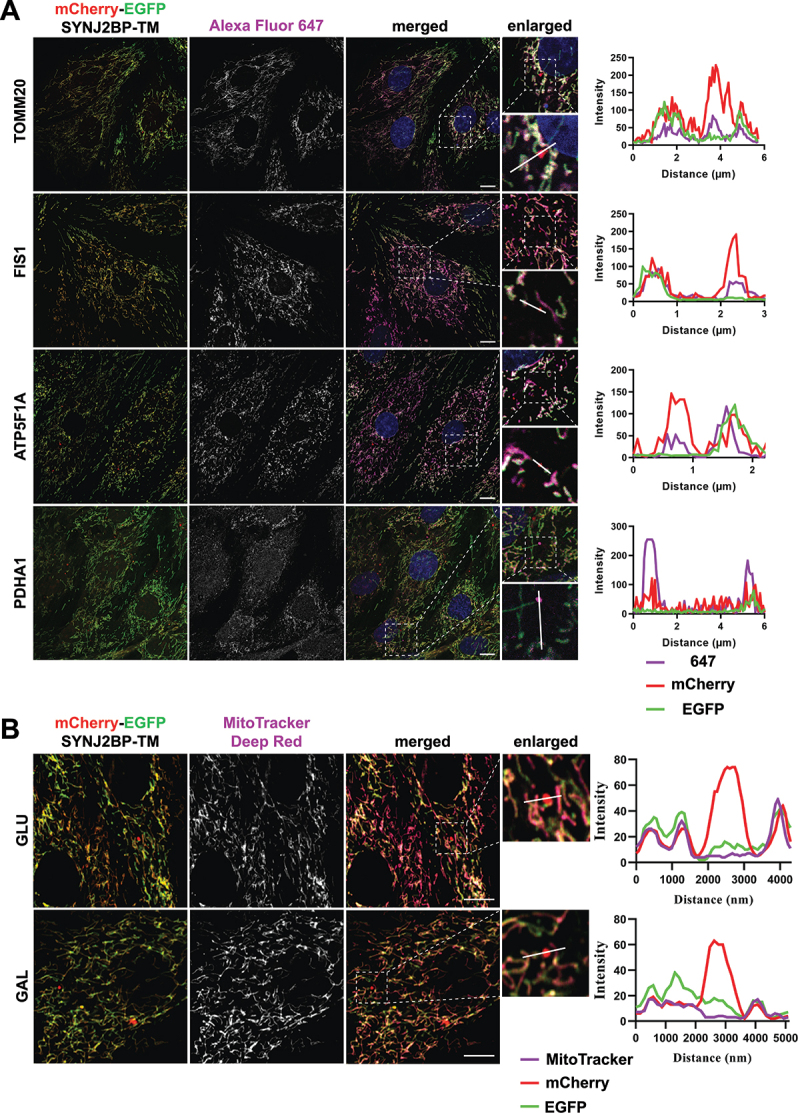
Red-only dots colocalize with mitochondrial markers of different mitochondrial compartments and lack membrane potential. (A) Confocal microscopy images of fixed tandem- tagged (mCherry-EGFP-SYNJ2BP-TM) H9c2 cells demonstrating colocalization of red-only dots with mitochondrial marker proteins of the different mitochondrial compartments; FIS1 and TOMM20 (outer membrane), ATP5F1 A (inner membrane) and PDHA1 (matrix). Region of interest for each marker is presented in a zoomed-in image with a line profile including an enlarged red-only dot displaying the colocalization. (B) Confocal microscopy images of live H9c2 cells with the mCherry-EGFP-SYNJ2BP-TM reporter and MitoTracker Deep Red staining for both glucose and galactose adapted cells. The enlarged region of interest depicts line profiles demonstrating colocalization of MitoTracker Deep Red in mitochondrial networks, but lack of colocalization in red-only dots. Scale bar: 10 μm.

To verify targeting of the mCherry-EGFP-SYNJ2BP-TM reporter to the mitochondria in H9c2 cells and to demonstrate that red-only structures contained other mitochondrial markers, we performed immunofluorescence staining with antibodies against the outer mitochondrial membrane proteins TOMM20 and FIS1 (fission, mitochondrial 1) as well as the inner membrane protein ATP5F1A/ATP5A (ATP synthase F1 subunit alpha) and the matrix protein PDHA1 (pyruvate dehydrogenase E1 alpha 1). As expected, our results showed a high degree of colocalization of the tandem-tagged reporter with all the different mitochondrial markers, indicating correct targeting of the reporter to the mitochondria (Figure 3A). In addition, several of the red-only structures stained positive for the markers of the different mitochondrial compartments, confirming the presence of mitochondrial proteins in addition to the reporter in an acidic environment (Figure 3A enlarged). For assessment of mitochondrial membrane potential, we incubated the cells with MitoTracker Deep Red and performed live confocal imaging of the cells. Interestingly, the red-only structures did not stain positive for MitoTracker Deep Red, indicating loss of membrane potential (Figure 3B). In summary, the red-only structures represent fragments of mitochondrial origin that have lost the membrane potential.

### Functional lysosomes give rise to red-only structures

**Figure 4.**
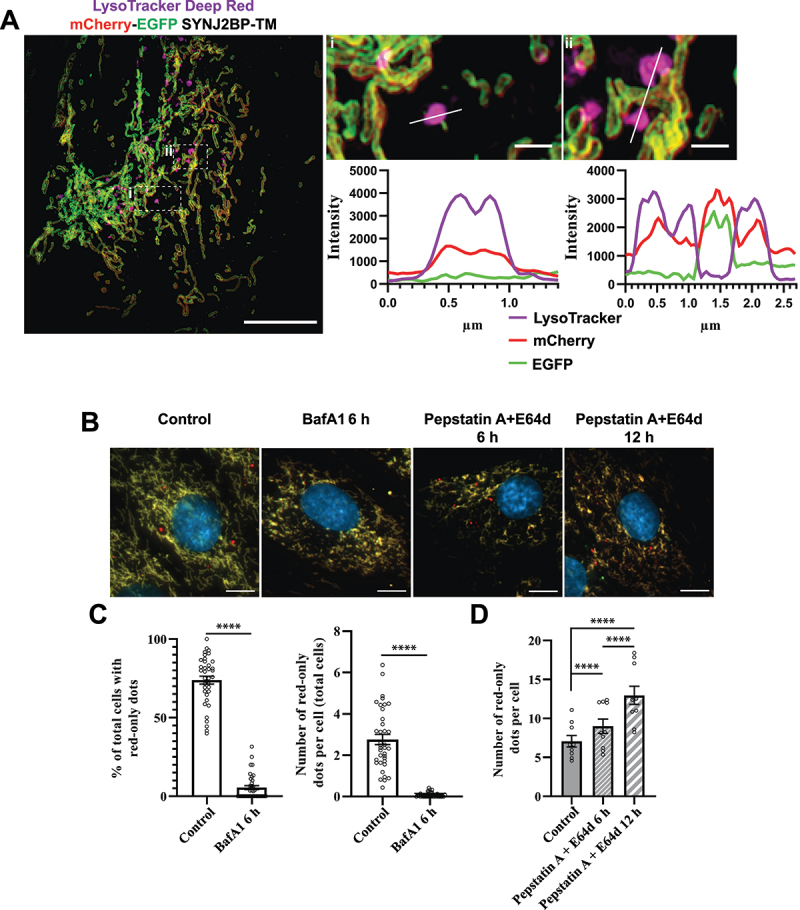
Functional lysosomes are essential for the appearance and removal of red-only dots during galactose adaption. (A) Structured illumination microscopy (SIM) imaging of fixed mCherry-EGFP-SYNJ2BP-TM H9c2 cells showing red-only dots that are positive for LysoTracker DeepRed staining (magenta). Line profiles through the LysoTracker-positive red-only dots in the enlarged boxed regions of interest are depicted, with corresponding numbers between the overview image and enlarged images. (B) Representative images of galactose adapted mCherry-EGFP-SYNJ2BP-TM H9c2 cells during control conditions and after treatment with the lysosomal inhibitors bafilomycin A_1_ (BafA1; 200 nM) and pepstatin a (PepA;10 μg/ml) and E64d (10 μg/ml) for the indicated times. (C) Quantification of the effects of a 6 h treatment of BafA1 on galactose adapted cells with the mCherry-EGFP-SYNJ2BP-TM reporter by assessing the percentage of cells containing red-only dots and number of red-only dots per total cells. (D) Quantification of the effects of a time course treatment of PepA and E64d assessed by number of red-only dots per cell in cells with red-only dots in galactose adapted cells with the mCherry-EGFP-SYNJ2BP-TM reporter. Over 150 cells were analyzed for each condition. The data is presented as mean ± SEM from 3 independent experiments, with more than 100 cells per condition. The individual datapoints are per frame cell averages. NOTE: * *p* < 0.05, ** *p* < 0.01, *** *p* < 0.001 and **** *p* < 0.0001. Scale bars: 10 μm and 1 μm (Ai and Aii).

To study colocalization of red-only mitochondria and acidic organelles (endo/lysosomes) we utilized LysoTracker Deep Red staining of the mCherry-EGFP-SYNJ2BP-TM H9c2 cells and applied three-dimensional (3D) structured illumination microscopy (SIM) on the cells after fixation. The obtained super-resolution images clearly demonstrated the presence of the reporter on the targeted mitochondrial outer membrane (Figure 4A). Furthermore, our results showed that most of the red-only mitochondria were positive for LysoTracker Deep Red staining, indicating colocalization of red-only mitochondria and lysosomes (Figure 4Ai,ii). To establish that the increased appearance of red-only mitochondria in galactose adapted H9c2 cells was in fact dependent on low pH inside lysosomes (or late endosomes) and that the reporter responded dynamically to lysosomal pH, we subjected the galactose adapted cells to bafilomycin A_1_ (BafA1) treatment for 6 h (Figure 4B). BafA1 inhibits the vacuolar type H^+^-ATPase (V-ATPase) and results in pH elevation in the lysosome lumen leading to a subsequent inhibition of resident hydrolases. In addition, BafA1 can impair fusion between autophagosomes and lysosomes [47]. As anticipated, the number of cells with red-only mitochondria as well as the number of these per cell was abolished in the presence of BafA1 (Figure 4C). In contrast, treatment with inhibitors of lysosomal cathepsins, pepstatin A (PepA) and E64d, does not affect acidification of lysosomes but hampers cargo degradation [47]. Indeed, treatment of the H9c2 cells with the cathepsin inhibitors for 6 or 12 h resulted in an increase in the number of cells with red-only mitochondria as well as the abundance of red-only dots per cell, demonstrating the importance of lysosomal cathepsins in degradation of red-only dots (Figure 4D). We also demonstrated the same effect of the lysosomal inhibitors in the H9c2 TOMM20-mCherry-EGFP cells (Figure S3). Taken together, these results indicate that functional lysosomes are a prerequisite for the appearance and degradation of acidic mitochondria.

### Capturing formation of acidic mitochondria by super-resolution imaging

**Figure 5.**
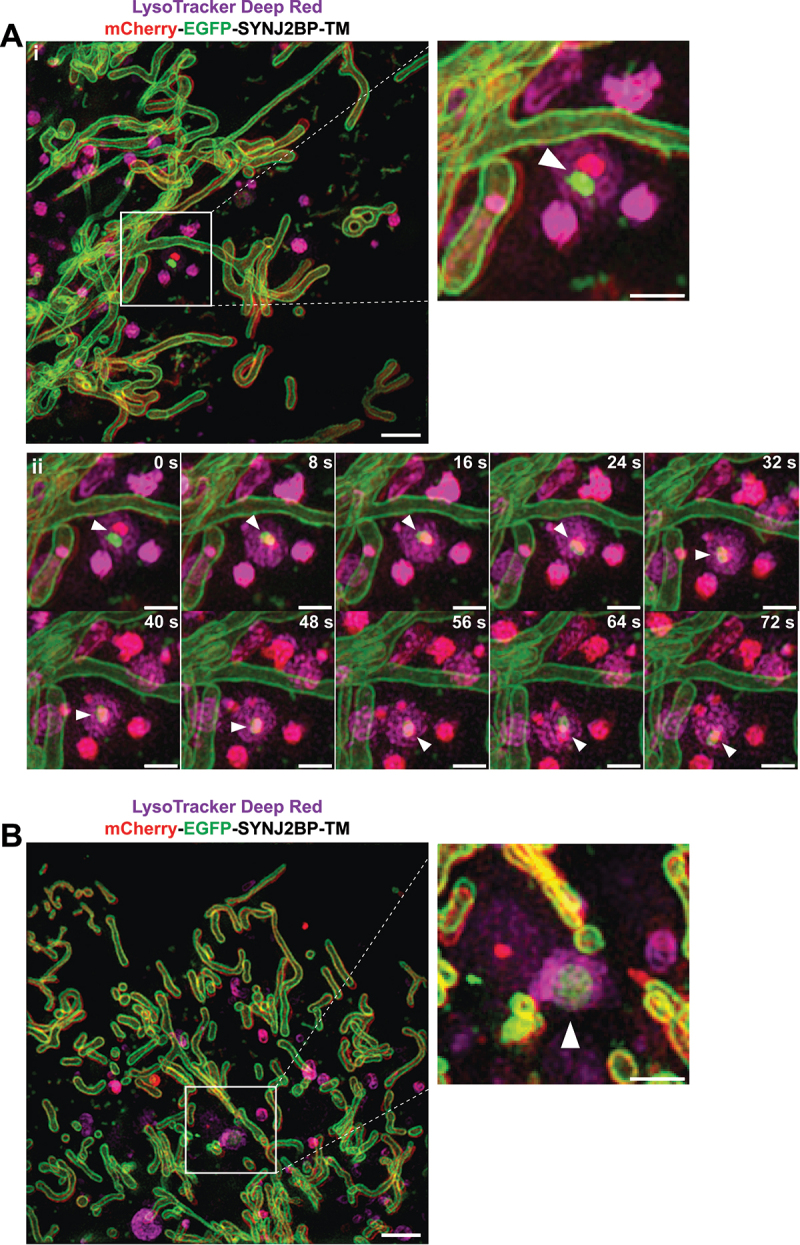
Three-dimensional (3D) structured illumination microscopy (SIM) live cell imaging of mCherry-EGFP-SYNJ2BP-TM H9c2 cells captures mitochondrial fragments within acidic structures. (Ai) A still frame overview image (merged channels) from the start of Video S1 displaying a cell during normal culture conditions after adding the LysoTracker Deep Red dye (100 nM for 40 min). The boxed area indicates a mitochondrial fragment with both EGFP and mCherry fluorescence inside a LysoTracker Deep Red-positive structure. The channels for red and green fluorescence are slightly shifted due to the time-delay between images of the different channels. The region of interest is shown as an enlarged image and the structure is highlighted with an arrowhead. (Aii) A time series of the boxed area in Video S1 following the movement of the lysosome containing the mitochondrial fragment (arrowhead). (B) A still frame overview image (merged channels) from the start of Video S2 displaying a cell after galactose adaptation after adding the LysoTracker Deep Red dye (100 nM for 40 min). The boxed area indicates a mitochondrial fragment with both EGFP and mCherry fluorescence inside a LysoTracker Deep Red-positive structure. The region of interest is shown as an enlarged image with the identified structure highlighted with an arrowhead. Max projection was utilized for all the images. Scale bars: 2 μm (overview images) and 1 μm (the enlarged images and for the time series).

**Figure 6.**
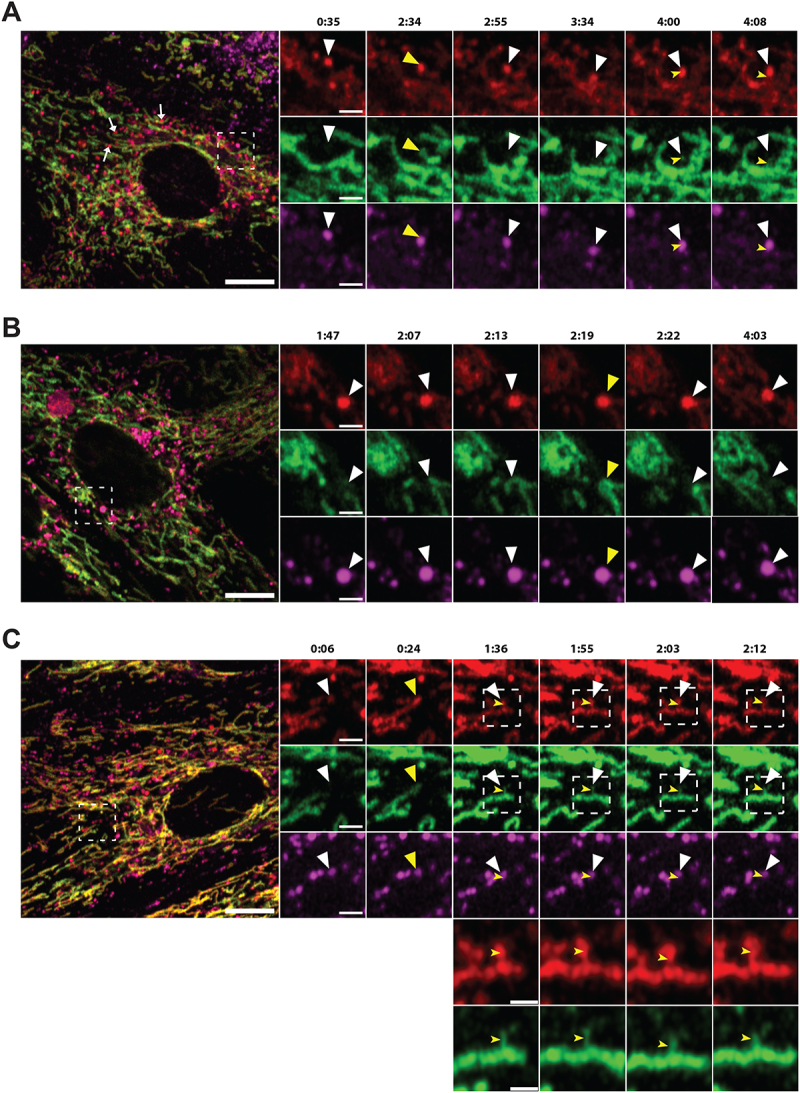
Airyscan FAST imaging of live galactose adapted mCherry-EGFP-SYNJ2B-TM H9c2 cells stained with LysoView 650 reveals transient contact and transfer between the mitochondrial network and red-only dots. (A) Full frame of video S3 at time 0:35 with zoomed-in area indicated (white box) for the selected time points (small panels, right). White arrows in the full frame show examples of red-only dots in close proximity with tubular mitochondria. White arrowheads in the small panels highlight a red-only dot approaching and contacting the mitochondrial network, producing a brief colocalized signal (yellow arrowheads) around time 2:34. At time 2:55, EGFP fluorescence has diminished, and the structure again appears red-only (white arrowheads). In subsequent frames, small protrusions of EGFP fluorescence (notched yellow arrowheads at time points 4:00 and 4:08) can be seen upon close inspection to extend from the mitochondrion and into the red-only dot, and then rapidly disappear (see also video S3). (B) Full frame of video S4 at time 1:47 with zoomed-in area indicated (white box) as in (A). White arrowheads indicate a red-only dot approaching the mitochondrial network from time point 2:07. The EGFP signal remains detectable within the red-only dot for a few seconds around time 2:19 (yellow arrowheads) and then disappears around time point 2:22 (see also video S4). (C) Full frame of video S5 at time 0:06 with zoomed-in area indicated (white box) as in (A) and (B). White arrowheads indicate a red-only dot which encounters a tubular mitochondrion from time point 0:24 (yellow arrowheads). In subsequent frames, small protrusions of EGFP fluorescence can be seen to extend into the red-only dots (notched yellow arrowheads at time 1:36, 1:55, 2:03, and 2:12). An enlarged partial view (white boxes) of the mCherry and EGFP channels is shown below each time point. Scale bars: 10 μm (full frames), 2 μm (small panels), and 1 μm (enlarged views).

To visualize the formation of acidic mitochondria we performed three-dimensional (3D) structured illumination microscopy (SIM) of live mCherry-EGFP-SYNJ2BP-TM H9c2 cells during normal culture conditions and in galactose adapted cells after LysoTracker Deep Red staining. Interestingly, in live videos from cells in both growth conditions (Video S1 and S2) we could detect a LysoTracker-positive structure containing a mitochondrial fragment of around 500 nm with both red and green fluorescence, indicating recent engulfment since the EGFP fluorescence was still detectable (Figure 5A,B). We identified these structures in the start of the videos and thus were unable to trace their formation back in time (Figure 5A). The majority of LysoTracker positive structures however did not contain any traces of EGFP fluorescence, possibly reflecting their more “mature” or degradative state. Notably, there is a significant time-delay between capturing the different channels during live 3D SIM imaging resulting in a slightly shifted appearance of the red and the green fluorescence of the same mitochondrial structure (Figure 5A). This makes it challenging to follow the formation of red-only structures in live SIM videos. To overcome this issue, we stained cells with LysoView 650 and used Airyscan FAST imaging to record videos at high temporal and spatial resolution (500 frames, frame time 1.01 s), without time delay between channels (Video S3–S5). As expected, we saw an almost complete overlap between LysoView 650 and red-only dots, confirming their acidic nature. Furthermore, the highly motile red-only dots were observed in close proximity with tubular mitochondria (Figure 6A). The time-lapse imaging revealed the red-only dots engaged in numerous transient contacts with the tubular mitochondria (Figure 6A, 2:34; Figure 6B, 2:19; Figure 6C, 0:24) lasting from less than 30 seconds to several minutes. Following such contacts, a mitochondrion was in some instances seen to alter its shape to become notably pulled toward red-only dots (see mitochondrion in contact with two red-only dots in the enlarged view in Figure 6C; compare timepoints around 1:00 to 2:45 in video S5). Furthermore, we observed several instances of apparent rapid transfer of material between a mitochondrion and a red-only dot (Figure 6C enlarged views from 1:36 to 2:12). Importantly, in such cases EGFP fluorescence was only briefly detectable at the intersection between the structures, suggesting uptake into an acidic lumen and an almost instant quenching of the EGFP fluorophore. Taken together, by applying 3D SIM and Airyscan FAST live cell imaging we could capture lysosomal engulfment of mitochondrial contents.

### CLEM reveals red-only structures as single membrane vesicles containing collapsed mitochondria and lamellar lysosomes

**Figure 7.**
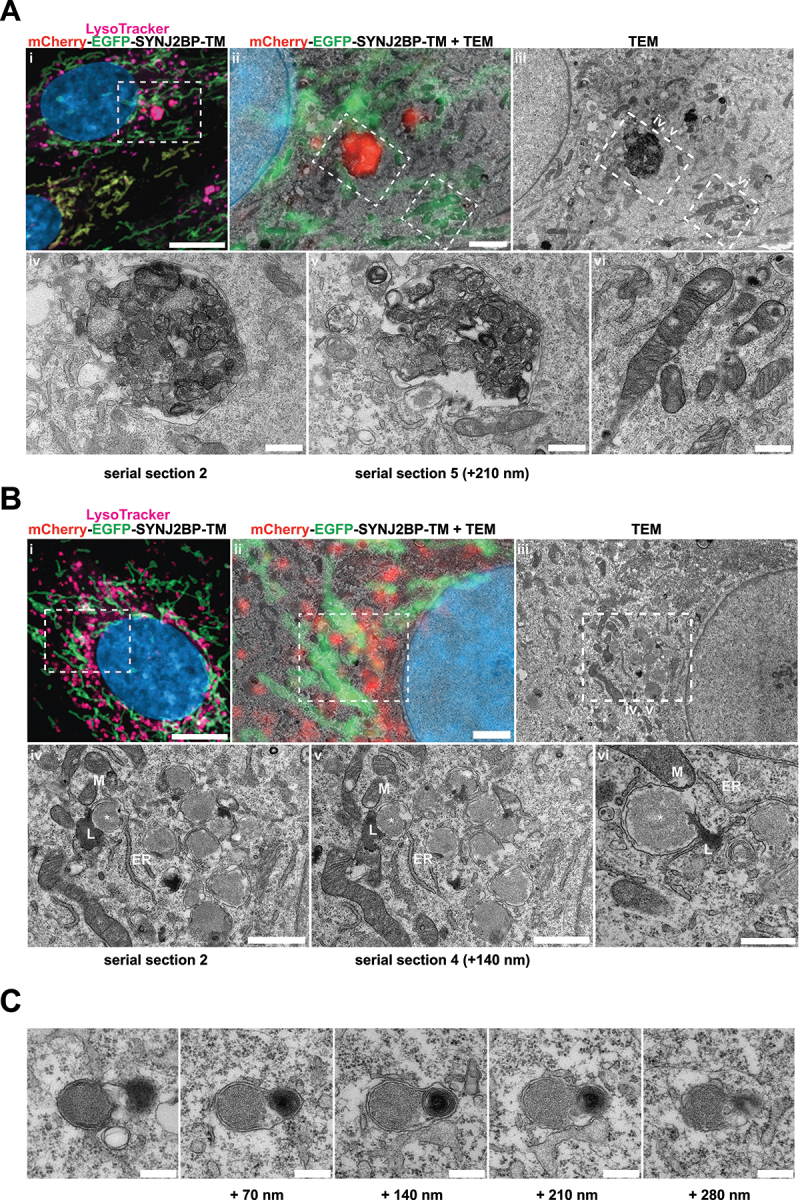
Correlative light and electron microscopy of mCherry-EGFP- SYNJ2BP-TM H9c2 cells reveals the ultrastructure of red-only dots. (A) Red-only dots in cells grown in media containing glucose correspond to autophagic vacuoles with a diverse internal milieu at varying stages of cargo engulfment and maturation (A iv-v). Meanwhile, mitochondria displaying both EGFP and mCherry fluorescence have a normal tubular morphology with distinct inner and outer membranes and intact cristae (A vi). (B) Structures corresponding to red-only dots in galactose adapted cells are more uniform and less electron dense. Imaging of consecutive ultrathin sections (B iv-vi) revealed that these structures are in close proximity (or may be continuous) with membranes of the endoplasmic reticulum (ER) and mitochondria (M). Also note apparent lysosomes (L) at the periphery of red-only structures (*). Scale bars: 10 μm (A i and B i), 2 μm (A ii and B ii), 1 μm (B iv and B v) and 0.5 μm (A iv-vi and B vi). (C) Serial section TEM imaging of a red-only dot shows a single membrane vesicle with apparent remnants of mitochondria and a multilamellar lysosome. Scale bar: 0.3 μm.

Correlative light and electron microscopy (CLEM) enables the determination of ultrastructural features of fluorescently labeled structures in a cellular context. For this type of high-resolution image analysis of the red-only dots we seeded our tandem-tagged SYNJ2BP-TM H9c2 cells grown under normal conditions or adapted to galactose on gridded dishes and stained the cells with LysoTracker Deep Red prior to fixation. Confocal imaging of the fixed cells and the grid after 4′,6-diamidino-2-phenylindole (DAPI) staining allowed us to relocate the coordinates of cells of interest after resin embedding. Serial section ultramicrotomy was performed on selected positions/cells, and the sections were then imaged by transmission electron microscopy (TEM). By overlay of confocal images and TEM images we could identify red-only mitochondria positive for LysoTracker Deep Red as quite electron dense structures with features typical of autophagic vacuoles, with varying size and content in cells under normal conditions (Figure 7A). The ultrastructural characteristics of red-only dots did not seem to depend significantly on their size, since smaller red-only dots showed similar features as larger ones (Figure S4A). In contrast, mitochondria with both EGFP and mCherry fluorescence had a tubular shape and normal cristae (Figure 7Avi). For the galactose adapted cells, the red-only structures were more homogenous and less electron dense (Figure 7B). Of note, when inspecting multiple consecutive sections of the same cells (Figure 7Biv-v), red-only structures were frequently seen in close vicinity (or in contact) with both the tubular mitochondrial network and the ER, as well as with electron-dense structures reminiscent of lysosomes (Figure 7Bvi). Serial section imaging revealed the ultrastructure of these red-only/LysoTracker-positive dots as single membrane vesicles surrounding remnants of what appeared to be collapsed mitochondria (no cristae) and an electron dense multilamellar lysosome (Figure 7C). To investigate the ultrastructure of red-only dots in a state of hampered lysosomal turnover, we performed CLEM after treatment with PepA and E64d, on cells grown in both glucose and galactose (Figure S4B and S4C, respectively). As expected, we observed an increased number of red-only dots which appeared heavily aggregated and had an increased electron density after lysosomal inhibition. Our results are consistent with increased lysosomal degradation of mitochondria after galactose adaptation and demonstrate the importance of imaging serial sections when characterizing ultrastructural features.

### Knockdown of *Ulk1, Atg7* or *Rab9a*, respectively, impacts on OXPHOS induced lysosomal degradation of mitochondria.

**Figure 8.**
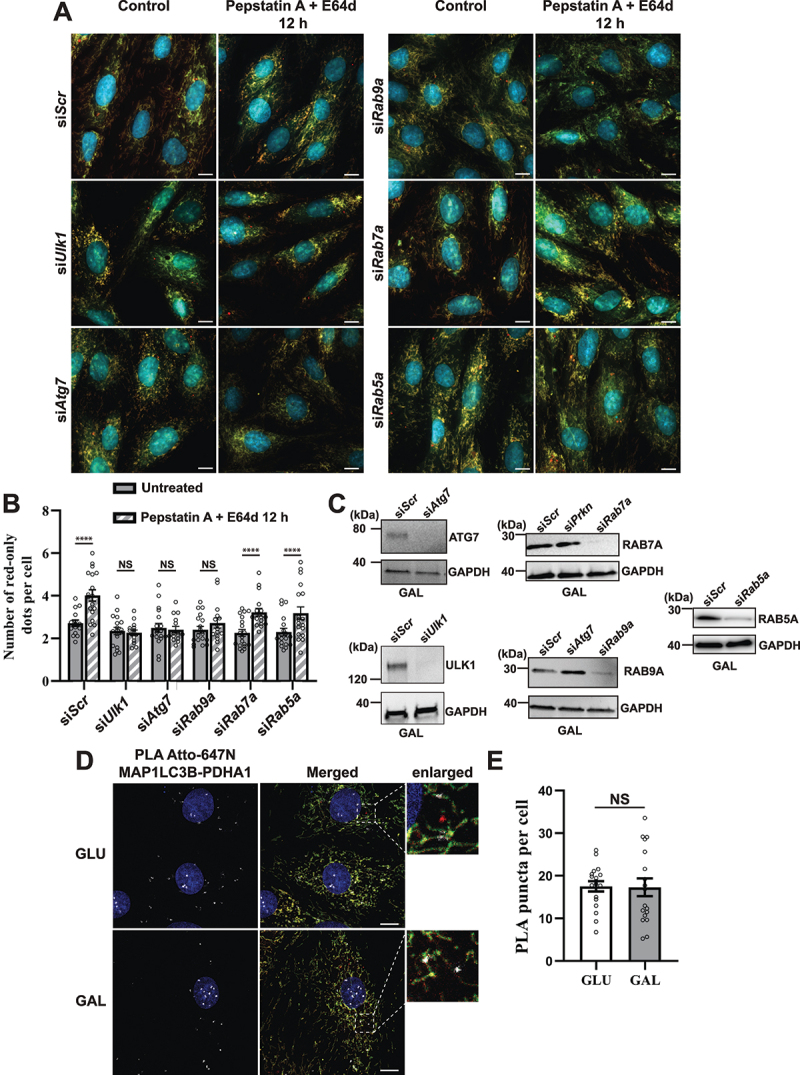
Effect of siRNA knockdowns on mitophagic flux and evaluation of LC3 involvement by proximity ligation assay (PLA). (A) Representative widefield images of mCherry-EGFP- SYNJ2BP-TM H9c2 cells analyzed 48 h after transfection with scrambled siRNA (siScr) or siRNA against *Ulk1*, *Atg7*, *Rab9a*, *Rab7a* or *Rab5a* respectively. (B) Quantification of the effect of 48 h siRNA knockdowns by assessment of number of red-only dots per cell in cells containing red-only dots in control conditions against a 12 h PepA and E64d treatment. The data is presented as mean ± SEM from 3 independent experiments, with more than 100 cells per condition (total number analyzed per experiment was over 1200 cells). (C) Western blots showing the expression levels of the siRNA targeted proteins in control and siRNA treated cells for verification of successful knockdown. (D) Representative confocal images of detected PLA puncta (white) using anti-MAP1LC3B and anti-PDHA1 antibody during normal (GLU) and galactose (GAL) adapted conditions. The enlarged boxes display the PLA puncta on the mitochondria network and small mitochondrial fragments but their absence on red-only dots. (E) Quantification of the number of PLA puncta per cell in 10 images (with more than 50 cells in total) per condition from two independent experiments. The individual datapoints are per frame cell averages. * *p* < 0.05, ** *p* < 0.01, *** *p* < 0.001 and **** *p* < 0.0001. Scale bar: 10 μm.

In an attempt to elucidate the mechanism(s) driving lysosomal degradation of mitochondria induced by galactose adaptation in H9c2 cells, we performed short interfering RNA (siRNA) knockdown of selected key genes involved in autophagic or endosomal degradation of mitochondria in cardiac cells: *Atg7* (canonical autophagy machinery) [48], *Rab9a* (alternative mitophagy) [28], *Ulk1* (upstream of both ATG7 and RAB9A) [15,28] as well as *Rab5a* (endosomal mitophagy) [27]. We also knocked down *Rab7a*, the small GTPase involved in direct contact between mitochondria and lysosomes [28]. We chose a knockdown strategy with siRNA due to difficulties in generating CRISPR-Cas9 knockouts in H9c2 cells as also previously reported [49]. The cells were subjected to 48 h of siRNA knockdown and grown with or without the lysosomal cathepsin inhibitors PepA and E64d during the final 12 h. After fixation, the cells were analyzed by fluorescence microscopy (Figure 8A). The use of PepA and E64d enabled us to assess perturbation of autophagic flux resulting from siRNA knockdown by quantifying red-only dots (Figure 8B). The knockdown of each protein was verified with western blots of cell lysates from cells adapted to galactose (Figure 8C). The abundance of red-only dots was quantified and compared with cells treated with PepA and E64d. Surprisingly, siRNA knockdown of neither *Ulk1*, *Atg7*, *Rab9a, Rab5a* nor *Rab7a* affected the number of red-only dots per cell during steady-state conditions. However, knockdown of *Ulk1, Atg7* and *Rab9a* led to an impaired flux, demonstrated by a lack of an increase in red-only dots after PepA and E64d treatment (Figure 8B). This did not apply to siRNA knockdown of *Rab5a* or *Rab7a* where the flux was unaffected. Our data thus show the importance of using lysosomal inhibitors when evaluating the effects of siRNA knockdowns on OXPHOS induced degradation of mitochondria. Prolonging the PepA and E64d treatment from 12 to 24 h did not uncover any further increase in the level of red-only dots (Figure S5A). Likewise, extending the siRNA knock-down to 72 h did not alter mitochondrial protein expression levels (Figure S5B). To further investigate the importance of the canonical autophagy machinery we monitored the presence of the autophagy marker MAP1LC3B on mitochondria and on red-only dots. To this end, we performed a proximity ligation assay (PLA) [50] using anti-MAP1LC3B antibody in combination with anti-PDHA1 antibody. This assay enables the assessment of proximity of the two targeted proteins in situ in fixed cells, giving rise to a fluorescent PLA signal or puncta only when the targeted proteins are within 40 nm of each other. The PLA puncta were localized on mitochondria, both networks and smaller structures, but were not present on red-only dots (Figure 8D). Notably, there was no increase in the number of PLA puncta per cell in galactose adapted cells compared to cells in normal glucose containing conditions (Figure 8E). This suggests the presence of MAP1LC3B on mitochondria before acidification but also indicates that MAP1LC3B is not the main mediator of enhanced mitochondrial degradation in galactose adapted cells. In conclusion, our data indicate the involvement of both ULK1, ATG7 and RAB9A in lysosomal degradation of mitochondria in H9c2 cells. In contrast, RAB5A and RAB7A do not seem to play a major role.

## Discussion

Given the central role of mitochondria in cell homeostasis, maintaining functional mitochondria is crucial. Basal levels of mitophagy have been considered too low for a reliable assessment [47]. Interestingly, use of pH-sensitive mitochondria reporters such as mt-Keima and *mito*-QC reveal a substantial but heterogenous level of basal mitophagy in tissues of mice and flies [51–56] and a detectable level in *C. elegans* and zebrafish [57]. Since the fraction of mitochondria targeted for lysosomal degradation at any given time under normal conditions is likely small compared to the total pool of mitochondria within the cell, such sensitive reporters are crucial to detect the degradation. The *mito*-QC reporter has been used in H9c2 cardiomyoblasts to display induced mitophagy during cell differentiation [49]. We exploited a similar dual color fluorescence-quenching assay based on a mCherry-EGFP outer mitochondrial membrane targeted reporter to detect lysosomal degradation of mitochondria in H9c2 cells. Our quantification of basal mitophagy in H9c2 cells showed a high percentage (around 50%) of cells containing red-only mitochondria. This is substantially higher than reported for e.g., human neuroblastoma SH-SY5Y cells [58] and mouse embryonic fibroblasts [59,60] where less than 20% of the cells display red-only mitochondria. Notably, the number of acidic mitochondrial structures detected per cell (around 3–4) of H9c2 cells with red-only dots was similar as to that reported for SH-SY5Y cells and human retinal pigment epithelial ARPE-19 cells [61]. By employing a metabolic shift to OXPHOS by galactose adaptation we detected a significant increase in both the number of cells containing acidic mitochondria as well as the number of red-only dots per cell. Our results are in line with studies on OXPHOS-induced mitophagy in mouse endothelial fibroblasts (MEFs) [59] as well as in HeLa cells and human primary skeletal muscle myoblasts [62]. In addition, piecemeal mitophagy of specific mitochondrial proteins is OXPHOS induced in HeLa cells [63] and in MEFs [60]. Most likely the induced removal of mitochondria or mitochondrial proteins is due to higher turnover of the mitochondria during elevated activity of the electron transport chain. This would sustain renewal of mitochondria and avoid accumulation of damaged organelles. In support of this, we have previously shown an increased formation of mitochondria derived vesicles in galactose adapted H9c2 cells [35]. Conversely, OXPHOS dependance blocks iron chelator-induced mitophagy in human bone osteosarcoma U2OS cells and SH-SY5Y cells [58] and Carbonyl cyanide 3-chlorophenylhdyrazone (CCCP) depolarization-induced mitophagy in neurons and HeLa cells [64–67]. Hence, the metabolic status of the mitochondria can dictate the level of mitophagy, dependent on the cell type and growth conditions. Interestingly, a recent study suggests that mitophagy itself initiates an increase in mitochondrial biogenesis and oxidative metabolism in induced pluripotent stem cells undergoing endothelial differentiation [68].

Using a deep-learning approach we were able to quantify the morphological change of mitochondria in H9c2 cells, adopting a more fragmented or shorter appearance after the metabolic shift. Mitochondrial fragmentation has also been observed in neonatal cardiomyocytes where the percentage of mitochondria with shorter lengths increases during glucose deprivation by galactose adaptation [28]. Mitochondrial fragmentation is often mediated by peripheral fission which is mechanistically different from mid-zone fission directing mitochondria biogenesis [42]. In green monkey kidney fibroblast-like Cos-7 cells the smaller peripheral fission-derived mitochondria display a length distribution of only 1–2 μm and are mainly subjected to degradation. In addition, the rate of peripheral fissions per cell increases when the Cos-7 cells are grown in glucose-free, galactose containing media [42].

Performing super-resolution live cell imaging on tandem tagged SYNJ2BP-TM H9c2 cells with labeled lysosomes, we were able to monitor highly dynamic interactions between lysosomes and mitochondria. Furthermore, we detected rapid lysosomal engulfment of mitochondrial contents within a few minutes. The rapid formation of these structures could also indicate their rapid degradation and thus influence the numbers of such events detected in snapshots of fixed cells. The potential consequence could therefore be an underestimation of the levels of lysosomal degradation of mitochondria when studying fixed cells and tissue. We are not aware of previous publications demonstrating super-resolution live cell imaging of the formation of acidic mitochondria.

Applying CLEM, we revealed the ultrastructure of red-only mitochondria in the H9c2 cells in steady-state conditions and after a metabolic shift toward OXPHOS. There are only a few studies that have performed CLEM analysis on acidified mitochondria. The ultrastructures of mt-Keima acidic dots analyzed by CLEM in *Drosophila* muscle cells also contain features of multilamellar bodies and are of a comparable size as those detected in the H9c2 cells [55]. In studies performed in mammalian cells, iron depletion with DFP [57,67] or stress induced with propionic acid [69] or CCCP and overexpression of PRKN [70] are used to induce degradation of mitochondria before the CLEM analysis. Thus, to our knowledge, our study is the first to visualize the ultrastructure of acidified mitochondria in mammalian cells cultured under physiologically relevant conditions. Taken together, in view of our results from live cell SIM and Airyscan FAST imaging, the CLEM data presented are consistent with a model where mitochondria are fragmented at (or in close vicinity to) the ER, and rapidly fuse with lysosomes.

There are still many unanswered questions regarding the molecular mechanisms of basal mitophagy. Notably, pH-dependent mitochondrial reporters indicate acidification of tagged mitochondria or parts of mitochondria and thus their presence in acidic late endosomes or lysosomes. However, the route of the labeled mitochondria toward lysosomes is not revealed by the reporters. We chose siRNA knockdown of *Ulk1*, *Atg7*, *Rab9a* to assess the contribution of both canonical and non-canonical autophagy in lysosomal degradation of mitochondria in the H9c2 cells. Knockdown of neither *Ulk1, Atg7* nor *Rab9a* reduced the level of red-only dots in galactose adapted cells. However, these siRNA experiments resulted in hampered mitophagic flux, revealed using lysosomal inhibitors. Therefore, our data indicate the presence of redundant mechanisms for lysosomal degradation of mitochondria in H9c2 cells where both the canonical autophagy machinery and alternative RAB9A mediated mitophagy operate during OXPHOS reliant conditions. In addition, because of the unaffected basal level during all knockdown experiments, other mechanisms such as different types of micromitophagy [26] could be involved and require further investigations.

Our study shows that assessment and visualization of lysosomal degradation of mitochondria at high temporal and spatial resolution is feasible during basal conditions. Our results indicate highly dynamic interactions and transfer of material between mitochondria and lysosomes and give important insights that are valuable for future studies and therapeutic targeting of mitophagy.

## Materials and methods

### Cell culture

Rat cardiomyoblast H9c2 cells (Sigma-Aldrich, 88092904) were cultured in high-glucose (4.5 g/L) Dulbecco’s Modified Eagle Medium (DMEM; Sigma-Aldrich, D5796) with 10% Fetal Bovine Serum (FBS) and 1% streptomycin/penicillin (Sigma-Aldrich, P4333). For glucose deprivation and adaptation to galactose, the cells were grown in DMEM without glucose (Gibco, 11966–025) supplemented with 2 mM L-glutamine (Sigma-Aldrich, G7513) 1 mM sodium pyruvate (Sigma-Aldrich, S8636), 10 mM galactose (Sigma-Aldrich, G5388), 10% fetal bovine serum (Sigma-Aldrich, F7524) and 1% streptomycin-penicillin (Sigma-Aldrich, P4333). The cells were adapted to galactose for at least 7 days before the experiments. Stable H9c2 cells (see below) were grown in the same medium with the addition of 1 μg/ml of puromycin (InvivoGen, ant-pr-1). For hypoxic conditions, the cells were incubated at 0.3% O_2_ for 2, 4, 6 or 16 h. For labeling of lysosomes, the cells were treated with 50 or 100 nM LysoTracker Deep Red (ThermoFisher Scientific, L12492) for 30–40 min or with LysoView 650 (Biotium, 70059). Cells were treated as indicated with 0.2 μM bafilomycin A_1_ (BafA1 from *Streptomyces griseus*; Sigma-Aldrich, B1793) or 10 μg/ml pepstatin A (Sigma-Aldrich, P5318) and 10 μg/ml E64d (Sigma-Aldrich, E8640). All cell lines were maintained at 37°C and under 5% CO_2_.

### Generation of stable mCherry-EGFP-SYNJ2BP-TM and TOMM20-mCherry-EGFP H9c2 cell lines

H9c2 cells with stable expression of tandem tagged (mCherry-EGFP) mitochondria

outer membrane protein SYNJ2BP/OMP25 (synaptojanin 2 binding protein)-transmembrane domain (TM) or tandem-tagged (C terminus) full length TOMM20 protein were generated by retroviral transduction. The mCherry-EGFP- SYNJ2BP-TM construct [32] was amplified with PCR and cloned into the retroviral expression vector pMRXIP with the selection marker puromycin. The vector was made with deletion of GFP-STX17 from the pMRXIP-GFP-STX17 plasmid (Addgene, 45909; Noburo Mizushima lab). Full-length TOMM20 was amplified by PCR using mTagBFP2-TOMM20-N-10 plasmid (Addgene, 55328; Michael Davidson lab) as a template and cloned into the pMRXIP vector with the tandem tag. The plasmids were verified by restriction enzyme digestion and DNA sequencing (Applied Biosystems, 4337455BigDye). The HEK293-Phoenix packaging cell line (ATCC, CRL-3213) was transfected with the pMRXIP-reporter vectors using MetafectenePro (Biontex, T040–1.0). The virus-containing media from transfected HEK293-Phoenix cells was harvested 24, 48 and 72 h post transfection. The harvested media was subsequently filtered through a 0.45-μm filter and then added onto subconfluent H9c2 cells. Hexadimetrinebromide-polybrene (Sigma-Aldrich, H9268) was added to a final concentration of 8 μg/ml. The H9c2 cells were incubated with the virus-containing media with polybrene for 6–12 h each time. The transduced H9c2 cells were then selected with 1 μg/ml of puromycin. Stable expression of mCherry-EGFP-SYNJ2BP-TM or TOMM20-mCherry-EGFP was verified by western blotting and confocal imaging. Furthermore, the cells were sorted by fluorescence activated cell sorting (FACS) to ensure approximately equal expression level of the mitochondrial outer membrane reporter.

### High-resolution respirometry

At the day of measurements, the mCherry-EGFP- SYNJ2BP -TM H9c2 cells, grown in glucose or adapted to galactose for 7 to 21 days, were trypsinized, and resuspended in their conditioned medium and counted using Countess II (ThermoFisher Scientific). Respirometry was performed in an Oxygraph-2k system (Oroboros Instruments, Innsbruck, Austria) calibrated to air (gain for oxygen sensor was set to 2) with standard cell culture medium at 37°C. The measurements were repeated in 4 independent experiments. Based on the cell number, a calculated volume of cells was added to the two stirred (750 rpm) chambers aiming to a final concentration of 0.4 × 10^6^ cells/ml. The cell counting was repeated to determine the exact cell concentration in each camber, and chambers were sealed to obtain a closed system. Analysis of the oxygen concentration in the chambers was performed using DatLab version 5.1.0.20 (Oroboros Instruments, Innsbruck, Austria). Decreasing oxygen concentration in the chambers resembled cellular oxygen consumption. When the oxygen consumption rate, OCR (O_2_ flux (pmolO_2_/s*ml)) reached a steady state level, a measurement was recorded displaying total cellular respiration (basal). Leak respiration was assessed by the addition of oligomycin (Sigma-Aldrich, O4876) in a final concentration of 5 µM. Subsequently, the proton gradient was released by stepwise titration (0.5 μM/step) of the uncoupler carbonylcyanide-3-chlorophenylhydra-zone (CCCP) (Sigma-Aldrich, C2759) until the maximum respiration was achieved (electron transport system capacity, ETS capacity). The addition of 0.5 μM rotenone (Sigma-Aldrich, R8875) an inhibitor of CI and 2.5 μM antimycin A (Sigma-Aldrich, A8674) an inhibitor of CIII blocked mitochondrial respiration completely, resulting in residual oxygen consumption (ROX). The respiration measurement at the different STATES (basal, leak, ETS) were corrected for ROX afterward. Mitochondrial respiration was calculated by subtracting the non-mitochondrial respiration after antimycin A addition from the basal respiration level. ATP linked respiration was derived from subtracting the leak respiration (oligomycin) from the basal level. All respiration data was normalized to the cell count in the camber. The results were presented as a mean.

### Mitochondrial ROS measurements with MitoSox Red

H9c2 cells grown under normal culture conditions or adapted to galactose were treated with 100 nM antimycin A for 4 h or left untreated. After treatment, cells were harvested by trypsinization, washed three times with respective media and then incubated with 1 μM MitoSOX Red (ThermoFisher Scientific, M36008) for 30 min inside the cell incubator in their respective media. The cells were then washed 3 times with HBSS (Gibco, 1402–092) followed by flow cytometry analysis with a LSRFortessa (BD Biosciences). The excitation light used was 488 nm, emission was passed through 556LP filter and detected using a 616/23 nm emission filter. For confocal imaging purposes the cells were seeded in MatTek dishes (MatTek, P35-1.5-14-C) treated with antimycin A and stained with MitoSOX Red as described above, followed by HBSS washing prior to live cell confocal imaging. MitoSOX Red fluorescence was excited using the 514 nm laser and the emitted light was detected between 565–715 nm.

### Mitochondrial membrane potential visualization

The mCherry-EGFP-SYNJ2B-TM H9c2 cells grown under normal conditions or adapted to galactose were incubated with MitoTracker Deep Red (ThermoFisher Scientific, M22426) at 100 nM concentration for 30 min. The cells were then given fresh cell culture media and subjected to live Airyscan FAST imaging using the Zeiss LSM 880.

### Antibodies

The following primary antibodies were used: anti-GAPDH (Sigma-Aldrich, G9545; 1:5000) anti-TOMM20 (Santa Cruz Biotechnology, SC-11415; 1:500), anti-FIS1 (Proteintech, 10956–1-1ap; 1:100), anti-ATP5F1A/ATP5A (Abcam, Ab14748; 1:200), anti-PDHA1 (Abcam, ab110330; 1:200), anti-ATG7 (Cell Signaling Technology, 8558; 1:1000), anti-RAB9A (Cell Signaling Technology, 5118; 1:1000), anti-ULK1 (Cell Signaling Technology, 8054; 1:1000), anti-RAB5A (Cell Signaling Technology, C8B1; 1:1000), anti-RAB7 (ERP7589; Abcam, ab137029; 1:1000), anti-LC3B (Sigma-Aldrich, L7543;1:200). Alexa Fluor 647‐conjugated goat anti-rabbit and anti-mouse IgG (Invitrogen, A21244 and A32728; 1:500) were used as secondary antibodies.

### Immunostaining of fixed cells

For immunofluorescent staining the cells were seeded on #1.5 glass coverslips. At

approximately 80% confluence the cells were subjected to treatment. The cells were then fixed using 4% formaldehyde (ThermoFisher Scientific, J19943.K2) at 37°C for 20 min. The cells were permeabilized with methanol at room temperature for 5 min. The permeabilized cells were blocked with 3% pre-immune goat serum (Sigma-Aldrich, G6767) in phosphate-buffered saline (PBS; Sigma-Aldrich, D8537) for 1 h at room temperature before overnight incubation at 4°C with primary antibodies diluted in PBS with 1% goat serum. Cells were then washed and incubated for 1 h with Alexa Fluor-coupled secondary antibodies diluted 1:500 in PBS supplemented with 1% goat serum. After a final wash in PBS the coverslips were mounted on glass slides using Prolong Glass (Invitrogen, P36980).

### RNAi

The short interfering RNAs (siRNAs) used were pre-designed and validated *Silencer*®Select siRNAs (Invitrogen, 4390771): siRNA against *Ulk1* (siRNA ID s166350), siRNA against *Atg7* (siRNA ID s161900), siRNA against *Rab9a* (siRNA ID s136762), siRNA against *Rab5a* (siRNA ID s134381), siRNA against *Rab7a* (siRNA ID s131440) and negative control siRNA (Ambion, 4390844). The cells were transfected with siRNA using Lipofectamine RNAiMax Transfection Reagent (Invitrogen, 13778–075) according to the manufacturer´s recommendation. After 6 h of incubation the cell media was changed to remove the transfection reagent in order to avoid H9c2 cell death. After 48 or 72 h (two consecutive siRNA transfections) of siRNA knockdown the cells were fixed or harvested for western blot analysis. The cells were fixed with 4% formaldehyde with 0.2% glutaraldehyde (Sigma-Aldrich, G5882). For each coverslip of fixed cells/condition, 10 positions (containing more than 100 cells in total) were selected and imaged as a Z-stack on a Cell Discoverer7 widefield microscope (Carl Zeiss Microscopy). Quantification of red-only dots was performed using the IMARIS imaging analysis software (see below).

### NaveniFlex Proximity Ligation Assay (PLA)

The PLA assay was performed according to Navinci’s recommendations using NaveniFlex MR In Situ Detection kit (Navinci, NF.100.2) and all incubations were performed in a humidity chamber. Briefly, cells were seeded on coverslips and grown until around 80% confluency. The cells were fixed using 4% PFA at 37°C for 20 min and permeabilized with 100% methanol at room temperature for 5 min. The coverslips were then washed two times in PBS, blocked with Blocking solution (Navinci, NF.1.100.01) for 30 min at 37°C and then incubated with two primary antibodies (derived from mouse and rabbit, respectively) diluted in primary antibody diluent (Navinci, NF.1.100.02) overnight at 4°C. As a negative control one coverslip was incubated in Antibody diluent with only one primary antibody. The coverslips were washed and then incubated with the PLA probes corresponding to the primary antibodies using Navenibody M1 (Navinci, NF.1.100.004,) and Navenibody R2 (Navinci, NF.1.100.05) in Navenibody Diluent (Navinci, NF.1.100.03) for 1 h at 37°C. Then, the coverslips were washed and subsequently incubated for DNA ligation with enzyme A (Navinci, NF.2.100.09) in buffer A (Navinci, NF.2.100.08) and enzyme B (Navinci, NF.2.100.11) in buffer B (Navinci, NF.2.100.10) for 1 hour and 30 min, respectively at 37°C. The coverslips were washed and finally incubated with enzyme C (Navinci, NF.2.100.15), a DNA polymerase, diluted in amplification buffer C Atto 647N (Navinci, NF.2.100.14) for 90 min at 37°C protected from light. Finally, the coverslips were stained with DAPI, washed, and then mounted on glass slides using Prolong Glass antifade mountant media. The fluorescent PLA signal and DAPI was detected using LSM800 confocal microscope (Carl Zeiss Microscopy) equipped with a 40X NA1.2 water immersion objective. Images were acquired as 3-slice Z-stacks. A minimum of 10 positions per coverslip of fixed cells (containing over 50 cells in total) were imaged per condition in two independent experiments. Quantification of PLA puncta was performed on the images using the IMARIS image analysis software.

### Western blot analysis of total H9c2 cell lysates

Cells were lysed by scraping in 2X sodium dodecyl sulfate (SDS) buffer (100 mM Tris-HCl, pH 6.8, 20% glycerol, 4% SDS) with 1× Complete Mini EDTA-Free Protease Inhibitor Cocktail (Roche, 11697498001) and boiling for 5 min. Total protein content of cell extracts was determined using a Bicinchoninic Acid (BCA) Kit (ThermoFisher Scientific, 23227). Total protein lysates (15 μg) were run on Mini-Protean TGX 4–20% gradient gels (Bio-Rad, 456–1093) and transferred onto InvitrolonTM PVDF membranes (Invitrogen, LC2005). Transfer was visualized with Ponceau staining and the membrane was blocked with 5% nonfat dry milk in TBST (20 mM Tris pH 7.5, 150 mM NaCl, 0.1% Tween 20 [P1379, Sigma-Aldrich]). The membrane was incubated with primary antibody overnight at 4°C followed by 1 h incubation at room temperature with horseradish peroxidase (HRP)-conjugated secondary antibody; BD Pharmingen HRP Goat Anti-Mouse Ig (BD Biosciences, 554002) or HRP-conjugated Affinipure Goat Anti-Rabbit IgG (H+L) (Proteintech, SA00001–2). Signal detection was performed with a western blotting chemiluminescent reagent (Sigma-Aldrich, CPS3500) and an iBright Imaging System (ThermoFisher Scientific).

### Widefield and confocal imaging of fixed cells

For imaging, Celldiscoverer7, LSM800 and LSM880 (all systems Carl Zeiss Microscopy) were used. For all images taken with the Celldiscoverer7 a Plan-Apochromat 50× objective with an NA of 1.2 was used. The images were acquired as z-stacks. The LSM800 was used with a Plan-Apochromat 63× oil (M27) objective with an NA of 1.4 for verification of colocalization of red-only dots and mitochondrial proteins. The Plan-Apochromat 40× water objective with an NA of 1.2 was used for imaging PLA puncta, which were acquired as z-stacks. For the LSM880 the images were acquired either with a Plan-Apochromat 63× oil objective with an NA of 1.4 or a C-Apochromat 40× water objective with an NA of 1.2. The LSM880 was used for bright field and fluorescence imaging in CLEM experiments to image cells of interest and to map the relevant grid coordinate for correlation with TEM images.

### Three-dimensional (3D) structured illumination microscopy (SIM) imaging of fixed and live cells cells.

The mCherry-EGFP-SYNJAB-TM H9c2 cells were seeded on MatTek dishes (MatTek Corporation, P35G-1.5-14-C) and imaged when they reached approximately 80% confluency. For labeling of lysosomes, the cells were treated with 100 nM LysoTracker Deep Red (ThermoFisher Scientific, L12492) for 40 min. After labeling, the cells were fixed with 4% formaldehyde and 0.2% glutaraldehyde or the media was replaced with fresh cell-culture media right before live imaging. The fixed cells were washed in PBS and imaged in PBS. For live cell imaging the cells were imaged in their growth medium at 37°C with atmospheric gas levels. The images were acquired using a DeltaVision OMX V4 Blaze imaging system (GE Healthcare) equipped with a 60X 1.42NA oil-immersion objective (Olympus), three sCMOS cameras, and 405, 488, 568, and 642 nm lasers for excitation. The vendor-specified optical resolution of the 3DSIM system is 110–160 nm laterally, and 340–380 nm axially, depending on the color channel. Super-resolution 3D images were obtained by 3DSIM reconstructions using the manufacturer-supplied softWoRx program (GE Healthcare). The SIM figure panels and AVI movies (maximum intensity z-projected and bleach corrected image sequenced using the exponential fit option) were assembled using Fiji [71].

### Airyscan FAST imaging of live cells after lysosome labeling with LysoView 650

Live mCherry-EGFP-SYNJ2B-TM H9c2 cells grown in MatTek dishes were stained with LysoView 650 (Biotium, 70059) for 30 min and imaged using the Airyscan FAST mode of the LSM880, utilizing line-wise switching between tracks to avoid time delay between channels. Cells were maintained in a humidified stage-top incubator at 37°C and 5% CO_2_ during imaging. Timelapse series (500 frames in total) were recorded using a 40× NA1.2 water immersion objective lens and a zoom factor of 4.0, with optimized scan settings for subsequent Airyscan processing (50 nm pixel size). The pixel dwell time was 0.73 μs, resulting in an individual frame time of 1.01 s. Laser excitation at 488 nm, 561 nm and 633 nm was used for EGFP, mCherry, and LysoView 650, respectively. The resulting raw files were processed using automatic settings (strength 6.0) in ZEN ver. 2.3 (Carl Zeiss Microscopy).

### Correlative-light and electron microscopy (CLEM)

Cells were grown on gridded #1.5 glass coverslips in 35-mm dishes (P35G-1.5-14-CGRD, MatTek). The cells were incubated with 50 nM LysoTracker Deep Red for 30 min before fixation with 4% formaldehyde and 0.5% glutaraldehyde in PHEM buffer, pH 6.9 (60 mM PIPES, 25 mM HEPES, 10 mM EGTA, 2 mM MgCl_2_). The cells were stained with DAPI and washed twice with PBS. After confocal imaging, the cells were processed for TEM using 0.05% malachite green (Sigma-Aldrich, 101398), 1% osmium tetroxide (Electron Microscopy Sciences, 19110)/0.8% K_3_Fe(CN)_6_ (Sigma-Aldrich, 702587), 1% tannic acid (Electron Microscopy Sciences, 21700), and 1% uranyl acetate (Electron Microscopy Sciences, 22400), followed by stepwise ethanol dehydration and embedding in epoxy resin (Agar, R1043). All processing steps were carried out using a microwave processor (Pelco BioWave, Ted Pella, Inc.). Finally, the resin was polymerized at 60º C for 48 h. After polymerization, the relevant dish coordinates were relocated and trimmed using a glass knife on an UC6 ultramicrotome (Leica Microsystems). Ultrathin sections (70 nm) were cut using a 35º ultra-knife (Diatome) and collected on slot grids. Sections were imaged using a HT7800 transmission electron microscope (Hitachi High-Tech) at 100 kV using a Xarosa CMOS camera and Radius ver. 2.0 (EMSIS). Preliminary image correlation of confocal images and TEM images was performed at the microscope using MirrorCLEM ver. 2.0.3 (Astron, Inc.). Final correlation was performed using the ec-CLEM plugin [72] in Icy ver. 2.4.2.0 [73] using DAPI, LysoTracker Deep Red, and tubular EGFP/mCherry-positive mitochondria as registration landmarks.

### Image analysis.

Fluorescence images were analyzed using Imaris ver. 9.6.1 (Bitplane). Images were converted from the .zen file format used by ZEISS microscopes into the .ims file format used by Imaris by the Imaris file converter. No preprocessing was performed. For the quantification of red-only mitochondria the Imaris XTension Channel Arithmetics was used in conjunction with an adapted Batch Processing XTension to create a new third image channel containing only areas where the mCherry signal was 50% higher than the EGFP signal. The Spots function was then

utilized to mark the mCherry signal and the DAPI-stained nuclei of all cells in the image, excluding those within 1 μm of the edge of the image. Each image was then manually inspected to remove artifacts. The Spots function was also utilized for the quantification of PLA puncta.

### Machine learning classification of mitochondria morphology

Classification of mitochondria was performed on the segmentation results obtained from the deep learning-based segmentation model that is trained on a simulated dataset [43]. The training dataset consists of thousands of images that are simulated by one, mimicking the geometrical shapes of mitochondria, and two, computationally modeling the process of image formation in a microscope. The simulated dataset is curated to closely match the microscope parameters of the data to be analyzed. The steps of segmentation began with the input confocal fluorescence images (EGFP-channel) that were cropped to sizes suitable for the deep learning-based segmentation model. The results from the segmentation model were then stitched back together to the original sizes of the images. The morphological classification of the individual mitochondria was done based on their branch lengths. For this, the segmentation results were first skeletonized using the Skan library [74] and the branch lengths of individual mitochondria were calculated for each experimental group. To prevent noisy segmentations from being included in the analysis, entries with branch lengths less than the resolution limit of the microscope were excluded. The rules for classifying mitochondria into the morphological classes of dots, rods, and networks were as follows; any mitochondria less than 1 µm in length was classified as a dot, those having lengths greater than 1 µm were further subdivided into rods, if they did not have junctions in their skeleton, and networks if they had at least one junction. The morphology classification was normalized per image frame for 19 or 22 images from each condition, glucose or galactose adapted cells respectively.

### Statistical analysis

The quantification data acquired using the IMARIS software underwent statistical analysis.

For the percentage of cells that contained red-only dots we approximated the proportions with normal distributions and thereafter we performed a two-tailed Z-test. For the average number of red-only dots per cell we assumed that these averages followed normal distributions so that a two-tailed Z-test could be utilized for the large sample sizes. The same was assumed for analysis of average number of PLA puncta per cell and for the morphological analysis. For the morphological analysis the statistical analysis was performed on a per frame basis. For the mitochondrial respiration data two-tailed paired t-tests were performed. Multiple comparison correction was performed with Bonferroni correction. Datasets subjected to quantitative and statistical analysis were from a minimum of three independent experiments, each independent experiment contained minimum 100 cells per condition analyzed. The exception was the dataset for PLA puncta quantification that was derived from two independent experiments and the morphological classification which had a dataset made from a single independent experiment. The PLA dataset contained minimum 50 cells per condition analyzed for each independent experiment, while the morphological classification was based on a single qualitative dataset of approximately 20 frames with representative cells from each condition. The data in graphs is represented as the mean ± SEM, individual datapoints are per frame averages. Statistical significance in the form of p-value is shown as * *p* < 0.05, ** *p* < 0.01, *** *p* < 0.001 and **** *p* < 0.0001.

## Supplementary Material

Supplemental Material

## Data Availability

The 3D SIM data that support the findings of this study are openly available in *Opstad, IS. et al, 2021, “3DSIM data of mitochondria in the cardiomyoblast cell-line H9c2 adapted to either glucose or galactose”, at https://doi.org/10.18710/PDCLAS, DataverseNO, V2*
