# Supplementary material for: High-resolution visualization and assessment of basal and OXPHOS-induced mitophagy in H9c2 cardiomyoblasts
Source: Autophagy. 2023 Jul 5;19(10):2769–88. doi: 10.1080/15548627.2023.2230837 (PMC10472865; doi:10.1080/15548627.2023.2230837)
Supplement: Supplemental Material [file KAUP_A_2230837_SM0387.zip › Supplemental Material_Godtliebsen et al._final R4.docx]

**Supplementary Materials**

**High-resolution visualization and assessment of basal and OXPHOS-induced mitophagy in H9c2 cardiomyoblasts**

Gustav Godtliebsen, Kenneth B. Larsen, Zambarlal Bhujabal, Ida S. Opstad, Mireia Nager, Abhinanda R. Punnakkal, Trine B. Kalstad, Randi Olsen, Trine Lund, Dilip K. Prasad, Krishna Agarwal, Truls Myrmel and Asa B. Birgisdottir^*^

***Corresponding author:**

Asa B. Birgisdottir, e-mail: aasa.birna.birgisdottir@uit.no

**This file includes:**

Figures S1-S5 with legends

Short description of Videos S1-S5

Materials and Methods description for mt-Keima mitophagy analysis


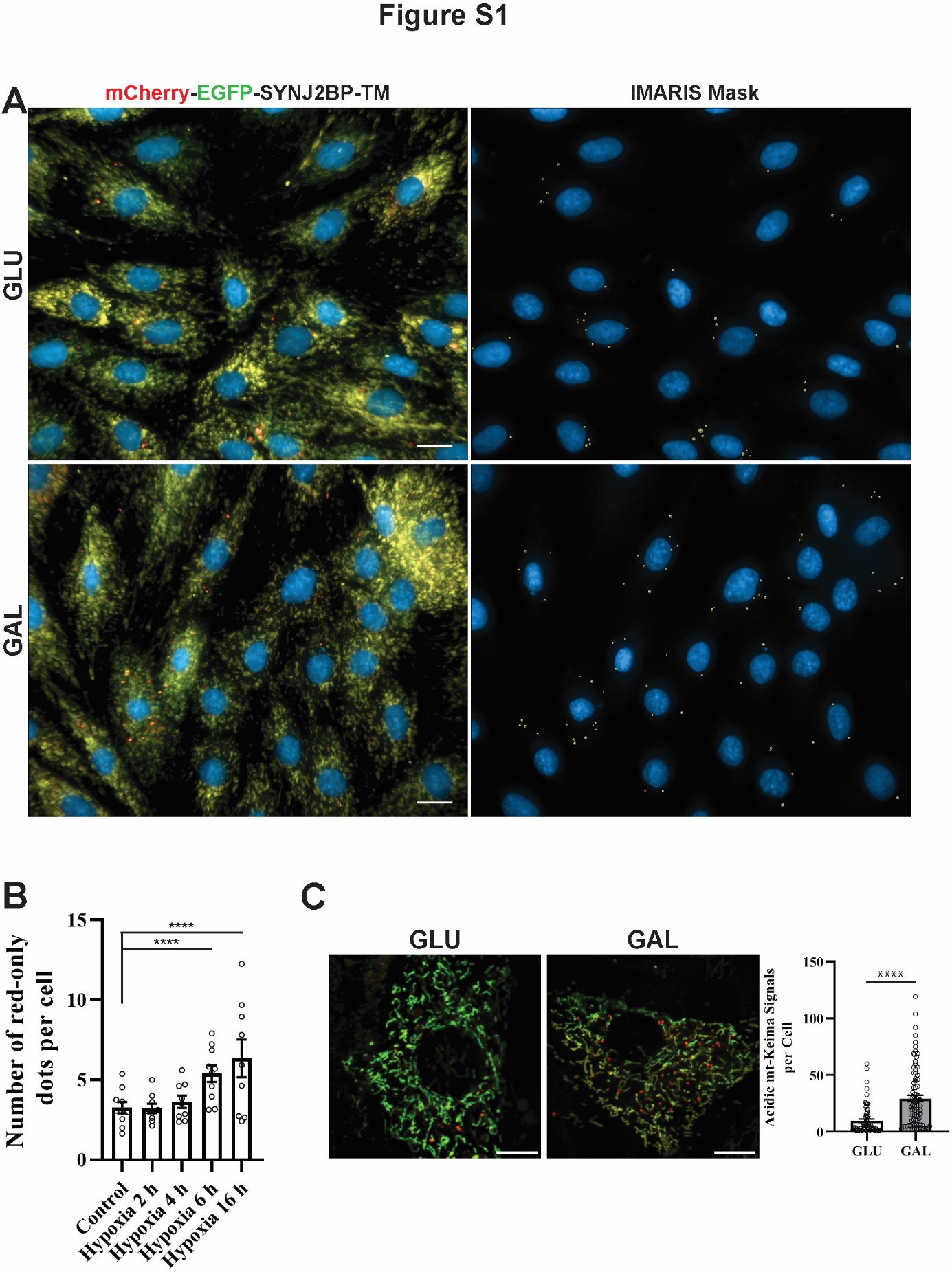


**Figure S1**. Detection of red-only dots applying the IMARIS image analysis software, evaluation of hypoxia time-course and mt-Keima as a model for mitophagy monitoring. (**A**) Representative widefield fluorescence microscopy images of H9c2 cells with a stable expression of mCherry-EGFP-SYNJ2BP-TM grown in normal glucose (GLU) conditions or adapted to galactose (GAL) media. The red-only dots identified and counted in each image employing the IMARIS software are depicted as an IMARIS mask. Nuclear DAPI staining was used for quantification of the number of cells per image. Scale bar: 20 µm. (**B**) The mCherry-EGFP-SYNJ2BP-TM cells were incubated under hypoxic (0.3 % O_2_) conditions for 2, 4, 6 or 16 h in normal media. As a control the cells were kept under normoxic conditions. Quantification of the number of red-only dots per cell in cells containing red-only dots during hypoxia. Results in graphs are shown as mean ± SEM of 10 fields of view containing number of cell (n), n ≥ 200, for each condition. (**C**) Representative images of mt-Keima transfected H9c2 cells in glucose media vs galactose adapted. The graph indicates the number of acidic mt-Keima signals per cell during the high glucose and galactose adapted condition. Scale bar: 10 µm. The individual datapoints are per frame averages for all graphs. * p<0.05, ** p<0.01, *** p<0.001 and **** p<0.0001.


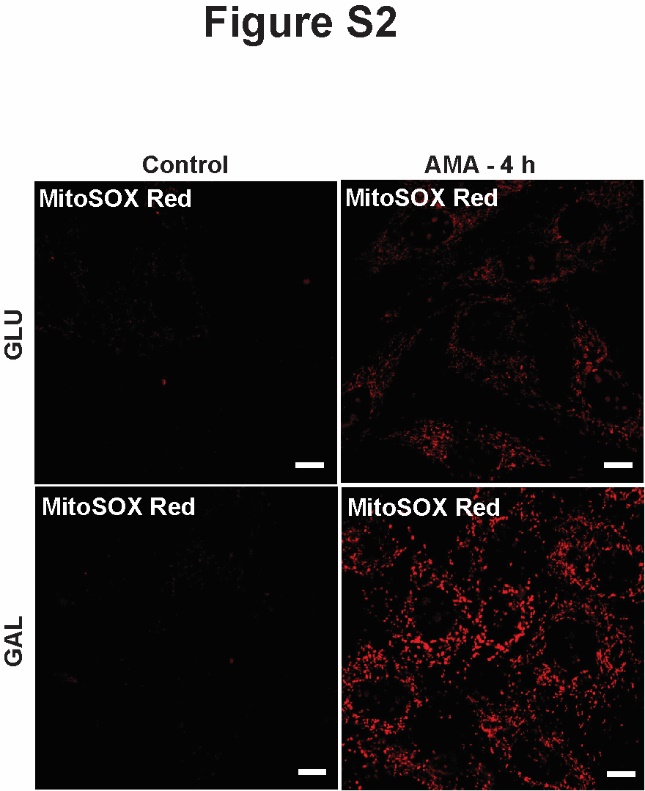


**Figure S2**. MitoSOX Red live cell imaging. Glucose or galactose adapted H9c2 cells were untreated or treated with 100 nM antimycin A (AMA) for 4 h, stained with MitoSOX Red and washed with HBSS. The MitoSOX superoxide indicator displayed higher intensity after AMA treatment compared to controls and was notably stronger in the galactose-adapted cells. Scale bar:10 µm.


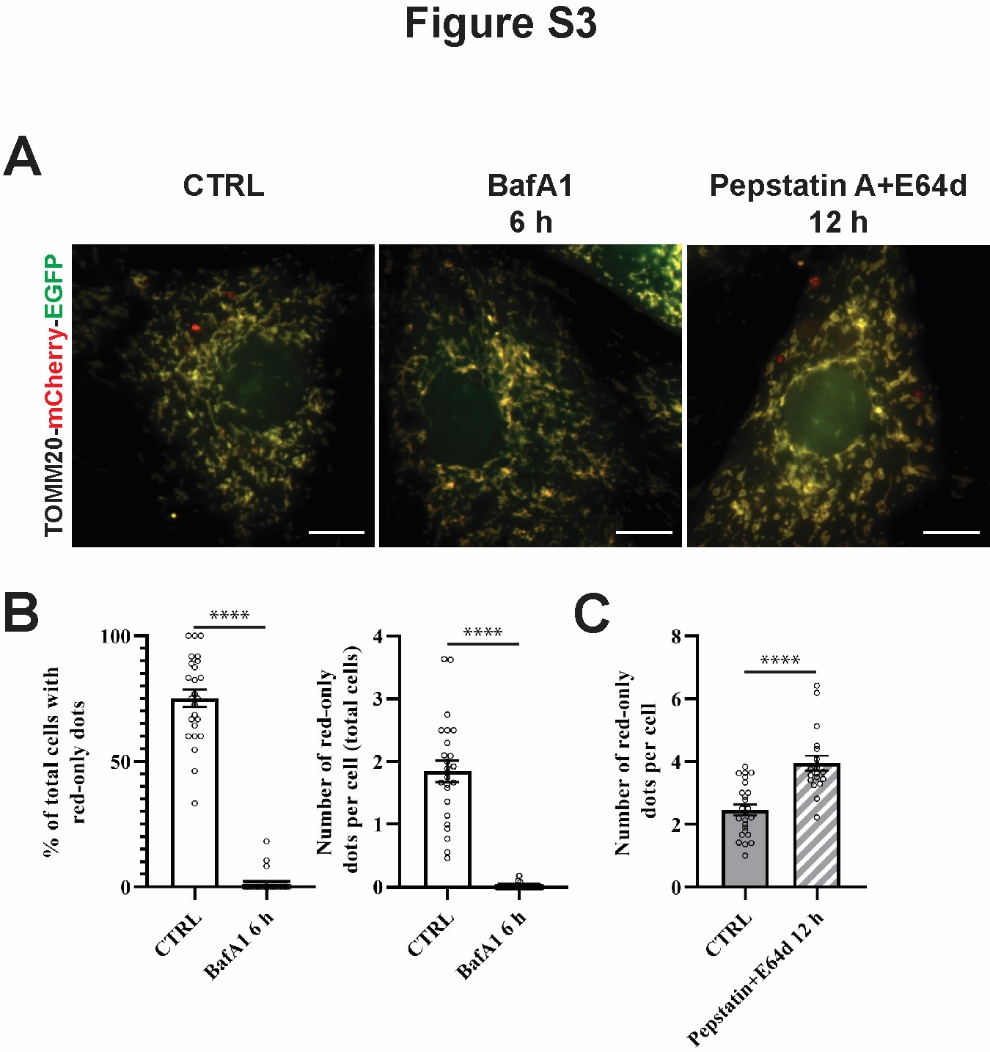


**Figure S3**. Evaluation of lysosomal inhibitors on the appearance and removal of red-only dots during galactose adaption. (**A**) Representative images of galactose adapted TOMM20-mCherry-EGFP H9c2 cells during control conditions and after treatment with the lysosomal inhibitors bafilomycin A_1_ (BafA1, 200 nM) or pepstatin A (10 µg/ml) and E64d (10 µg/ml), respectively for the indicated times. (**B**) Quantification of the effects of a 6-h treatment of BafA1 on galactose-adapted cells with the TOMM20-mCherry-EGFP reporter by assessing the percentage of cells containing red-only dots and number of red-only dots per total cells. (**C**) Quantification of the effects of a time course treatment of pepstatin A and E64d assessed by number of red-only dots per cell in cells with red only dots in galactose adapted cells with the TOMM20-mCherry-EGFP reporter. Data presented in (**B**) and (**C**) are shown as mean ± SEM from 3 independent experiments, with more than 100 cells per condition in each experiment. The individual datapoints are per frame averages. * p<0.05, ** p<0.01, *** p<0.001 and **** p<0.0001. Scale bar: 10 µm.


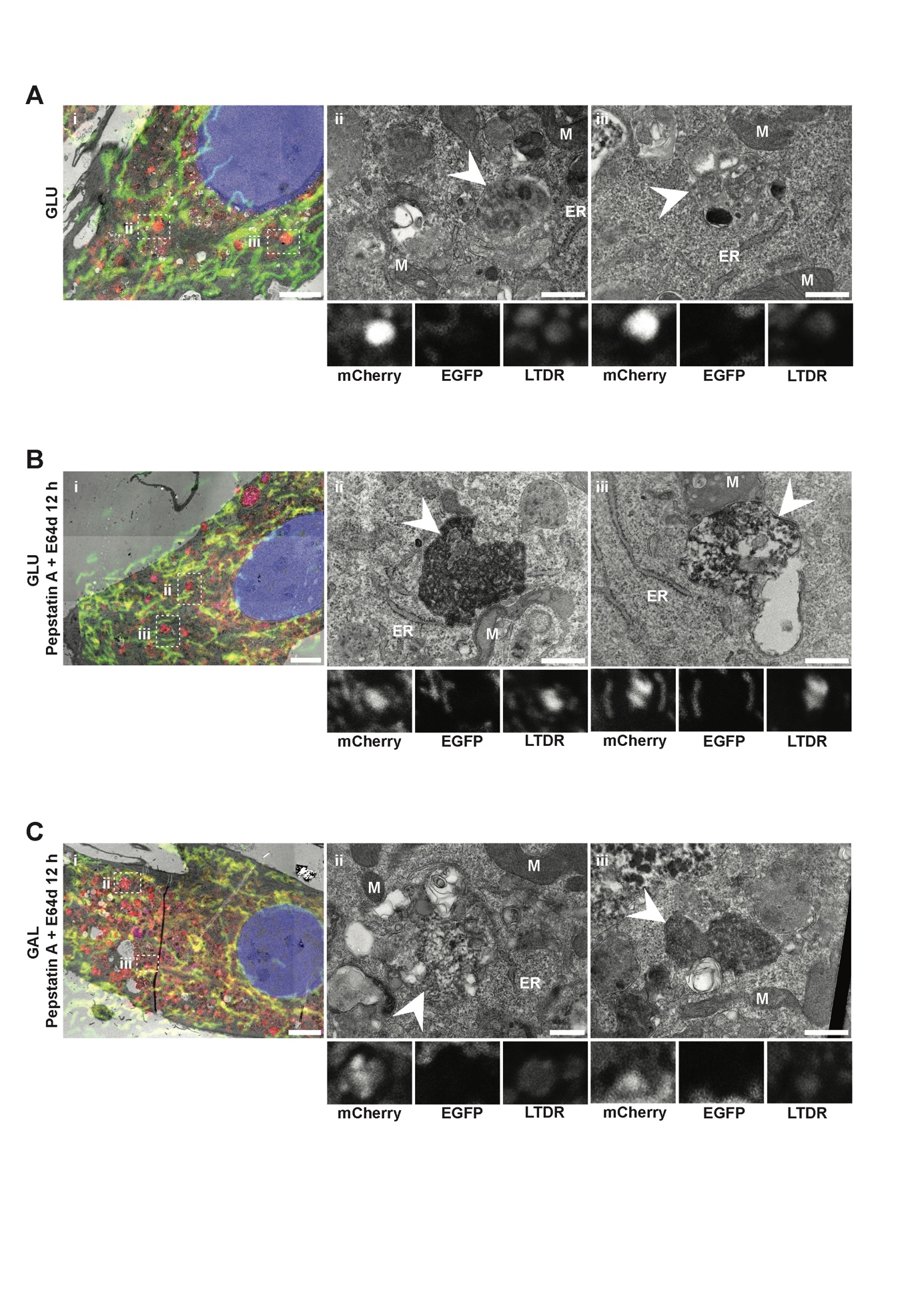


**Figure S4**. Correlative light and electron microscopy of mCherry-EGFP-SYNJ2BP-TM cells grown in glucose or grown in either glucose or galactose and treated with PepA and E64D. (**A**) In cells grown in glucose, small red-only dots (0.5–1 μm in diameter) do not show a significant difference in ultrastructure when compared to larger red-only dots (Fig. 7A). As for their larger counterparts, small red-only dots (arrowheads in Aii and Aiii) are found in close vicinity to tubular mitochondria and ER membranes and contain a diverse mix of engulfed material. Scale bars: 5 μm (Ai) and 500 nm (Aii and Aiii). Individual fluorescence channels corresponding to the TEM fields shown in Aii and Aiii are displayed below each panel. (**B**) and (**C**) In cells treated with PepA and E64D, red-only dots (arrowheads in Bii, Biii, Cii, and Ciii) are also found in the vicinity of tubular mitochondria and ER membranes. However, we observed a clear increase in electron density and overall aggregation of engulfed material within structures corresponding to red-only dots after inhibition of lysosomal turnover. Scale bars: 5 μm (Bi and Ci) and 500 nm (Bii, Biii, Cii, and Ciii). Individual fluorescence channels corresponding to each TEM field are shown below each panel as in (A).


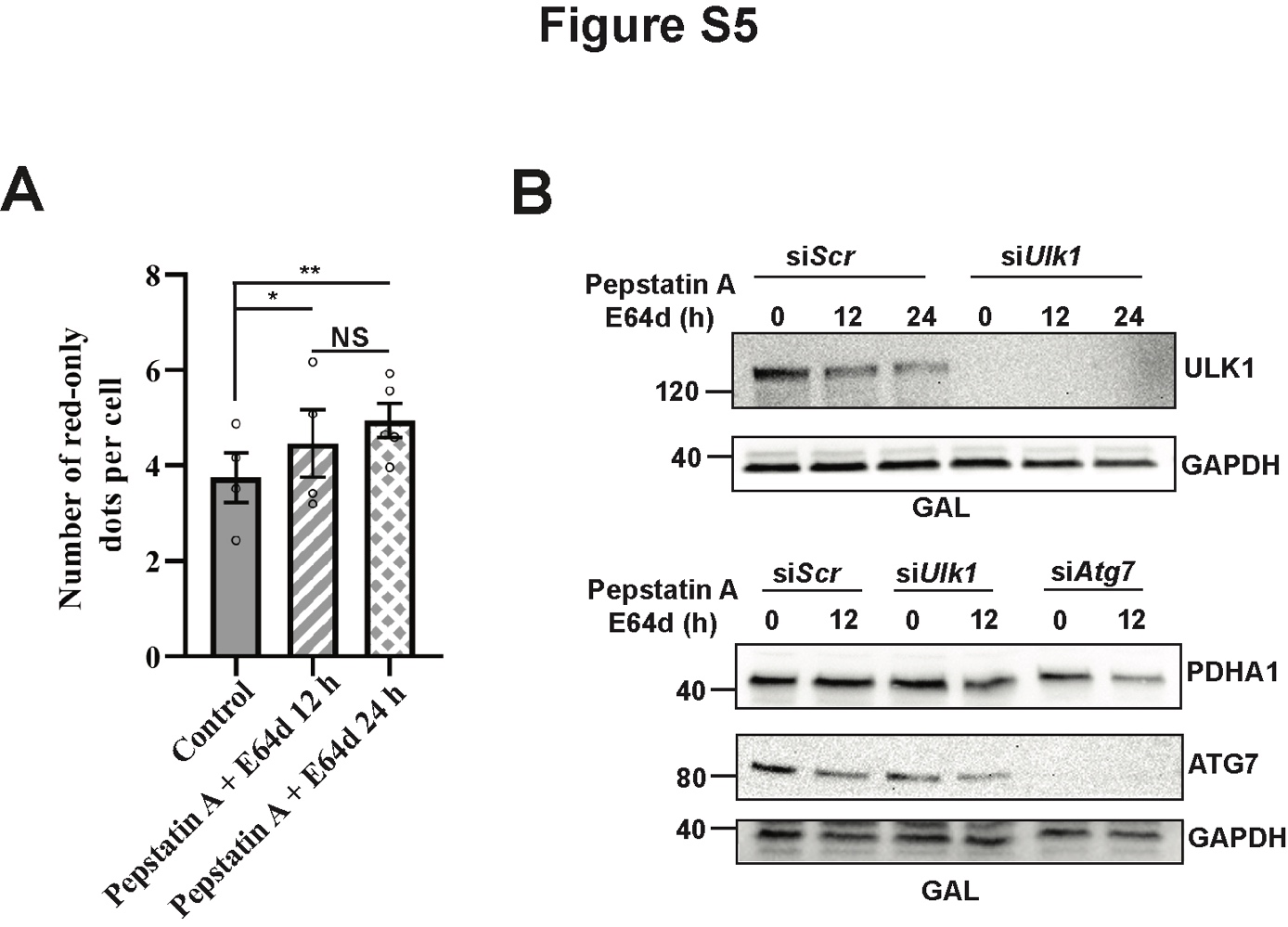


**Figure S5**. Assessment of extended treatments with pepstatin A and E64d for 12 and 24 h. (**A**) Quantification of the effects of a time course treatment of pepstatin A and E64d assessed by number of red-only dots per cell in cells with red only dots in galactose adapted cells with the mCherry-EGFP-SYNJ2BP-TM reporter. Over 100 cells were analyzed for each condition. The individual datapoints are per frame averages. (**B**) Western blot verification of the siRNA mediated knock down of the proteins ULK1 and ATG7, with indicated time of the pepstatin A and E64d treatment. * p<0.05, ** p<0.01, *** p<0.001 and **** p<0.0001.

**Video S1.** 3D SIM live cell video corresponding to the images shown in main Figure 5A (file name from data repository: 20210511_H9c2-dTag_GLU_LTDR100nm-40m_1520_sim256_10sTL_017_SIR_ALX_PRJ). Please note that the channels are colored here with mCherry as green, EGFP as blue, and LysoTracker Deep Red as red.

**Video S2.** 3D SIM live cell video corresponding to the images shown in main Figure 5B (file name from data repository: 20210511_H9c2-dTag_GAL_LTDR100nm-40m_1520_sim256_005_SIR_ALX_PRJ). Please note that the channels are colored here with mCherry as green, EGFP as blue, and LysoTracker Deep Red as red.

**Video S3.** Airyscan FAST live cell video corresponding to the images shown in main Figure 6A. mCherry (top left), EGFP (top right), and LysoView 650 (bottom left) panels are shown individually, while the merged panel (bottom right) shows mCherry and EGFP overlaid.

**Video S4.** Airyscan FAST live cell video corresponding to the images shown in main Figure 6B. Individual channels and the merged panel are as described for Video S3.

**Video S5.** Airyscan FAST live cell video corresponding to the images shown in main Figure 6C. Individual channels and the merged panel are as described for Video S3 and S4.

**Supplemental Materials and Methods**

***Assessment of mitophagy in H9c2 cells using mt-Keima***

For assessment of mitophagy with a matrix targeted pH-dependent probe, we transiently transfected H9c2 cells grown under normal conditions (high glucose) or adapted to galactose with mt-Keima plasmid mKeima-Red-Mito-7 (Addgene, 56018; Michael Davidson lab) using Neon Transfection System (ThermoFischer Scientific, MPK10096). The cells were analyzed 48 h post transfection by confocal microscopy. The fluorescence of mt-Keima was imaged in two channels via two sequential excitations (405 nm, green; 561 nm, red) and using a 570 to 695 nm emission range. Representative confocal images were processed manually. Calculation of mitophagy based on acidic mt-Keima signal was performed using the Zeiss ZEN software.
